# Enhancing Specific Stiffness of Nano‐Architected Materials Through Generative AI and Active Learning

**DOI:** 10.1002/advs.78140

**Published:** 2026-10-04

**Authors:** Jinwook Yeo, Peter Serles, Donggeun Park, Julia R. Greer, Seunghwa Ryu

**Affiliations:** ^1^ Department of Mechanical Engineering Korea Advanced Institute of Science and Technology (KAIST) Daejeon Republic of Korea; ^2^ Division of Engineering and Applied Science California Institute of Technology Pasadena California USA; ^3^ Department of AX Korea Advanced Institute of Science and Technology (KAIST) Daejeon Republic of Korea; ^4^ KAIST InnoCORE PRISM‐AI Center Korea Advanced Institute of Science and Technology (KAIST) Daejeon Republic of Korea

**Keywords:** active learning, generative AI, mechanical metamaterials, nano‐architected material, specific stiffness, variational autoencoder

## Abstract

Architected materials derive their high performance from geometry as much as from intrinsic material properties, yet most optimization frameworks search within predefined topology families, limiting the discovery of genuinely new morphologies. Here, we investigate whether superior architectures exist beyond the reach of these parameterized families, and demonstrate that they can be systematically discovered. We present a framework that searches directly in voxelated structural space. Cubic‐symmetric unit cells drawn from strut‐based, TPMS‐based, and hybrid families are encoded into a continuous latent representation, and surrogate‐guided multi‐objective search with an active learning loop repeatedly converts promising predictions into high‐fidelity validated data. Across nine rounds, the mean minimum Hamming distance from the initial dataset increases from 442 to 641 and the Pareto hypervolume rises from 0.106 to 0.113, confirming systematic expansion of the validated design domain. Computationally, the discovered architectures achieve 11–20% higher relative Young's modulus than initial designs at matched density. Fabricated via two‐photon polymerization and pyrolysis, the optimized carbon nano‐architectures outperform density‐matched counterparts by 18–23% in Young's modulus and 26–40% in strength, reaching specific stiffnesses of 17.2, 22.3, and 21.3 MPa m^3^ kg^−^^1^. These results establish that direct learning in structural space enables discovering mechanically efficient nano‐architectures beyond the initial parameterized families.

## Graphical abstract

A structure‐centric generative framework searches voxelated design space directly, coupling a variational autoencoder with multi‐objective optimization and active learning to progressively expand the validated design domain. The discovered nano‐architectures, realized as pyrolytic carbon by two‐photon lithography, deliver 18–23% higher stiffness and 26–40% higher strength than density‐matched benchmarks, reaching a specific stiffness of 22.3 MPa m^3^ kg^−^^1^.

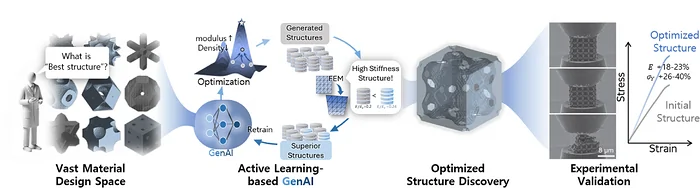

## 1 Introduction

Architected materials offer a route to mechanical performance that cannot be explained by intrinsic material properties alone. By distributing solid material in carefully designed 3D networks, shells, or bi‐continuous surfaces, these systems can achieve unusual combinations of stiffness, strength, recoverability, and energy absorption. This design freedom is especially important for lightweight engineering in aerospace, biomedical, and robotic systems, where the relevant performance metric is often specific stiffness. Recent advances in additive manufacturing have accelerated this progress, enabling the fabrication of intricate 3D architectures that were previously challenging to realize with conventional processes. For instance, previous studies which construct architectures at the nanoscale have demonstrated ultralight, ultra‐stiff mechanical metamaterials with exceptional stiffness‐to‐weight ratios [1, 2, 3]. 3D ceramic nanolattices exhibiting both high strength and recoverability have also been reported [4, 5, 6]. At small length scales, what was once a conceptual design problem has become a genuine materials‐discovery opportunity by combining efficient architectures with unique nanomaterial enhancements.

Despite these advances, conventional strut‐ and plate‐based lattices face persistent challenges. Nodes where members intersect introduce abrupt changes in cross‐section and curvature, concentrating stress under load and undermining yield performance; in severe cases they precipitate premature failure. Local geometric remedies have been explored to mitigate these issues, including the addition of fillets at stress‐prone joints, the incorporation of auxiliary support structures, and the modification of beam member profiles [7, 8]. However, improvements are typically incremental and strongly parameter dependent. Shell‐based architectures, particularly sheet‐type triply periodic minimal surfaces (TPMS), replace discrete nodes with smooth, continuous surfaces, thereby naturally mitigating node‐driven stress concentrations [9, 10, 11]. Even so, widely used TPMS families are instantiated from implicit level‐set equations with a small number of scalar parameters. The result is an enlarged but still strongly constrained manifold of candidate structures.

Optimization frameworks have long been used to improve architected designs. Topology optimization and related methods served as the primary tools for this purpose, but high‐resolution 3D applications remain computationally demanding, sensitive to regularization and initialization choices, and prone to convergence to local optima [12, 13]. Data‐driven optimization approaches, including Bayesian optimization and deep neural network (DNN) based inverse design, have therefore gained prominence [14, 15]. These methods have spurred notable advancements, with studies successfully demonstrating performance gains through the optimization of strut profiles, the investigation of TPMS‐based shell structures [16, 17, 18], and the combination of strut, plate, or shell elements [19, 20]. Despite these successes, a fundamental limitation persists: the exploration within these data‐driven frameworks is typically confined to predefined, low‐dimensional parameter spaces, for example, varying level‐set offsets or strut thicknesses. This reliance on parameter‐bound representations inherently restricts the discovery of truly novel structures. This representational constraint is most limiting when the goal is not to tune a known family but to discover whether a better architecture exists outside it. These limitations motivate the development of representations and search strategies that do not presuppose a specific analytical form.

Across these data‐driven approaches, whether the search operates on a parameter vector, a pretrained latent space, or a surrogate model of a fixed shape family, the representation is established before the search begins and remains fixed while it runs, so the search can reach only what that initial representation already encodes. Our own previous efforts likewise optimized parameters within fixed topology classes [6, 16]. A closely related framework was recently reported by Deng et al. [21], who compress a Gaussian‐process density‐field basis with a deterministic autoencoder trained once and then optimize a single objective at a fixed volume fraction in that fixed latent space. Our approach makes the complementary choice. The latent space is learned from FEM‐validated structures of several families, the search is multi‐objective and population‐based, and the generative model and the property surrogate are retrained on the newly validated candidates in every round, so that the representation itself evolves as the search expands beyond the initial data. The present work searches the structural space itself and addresses two questions, namely whether mechanically superior architectures exist beyond the reach of the known parameterized families and, if they do, whether they can be discovered systematically and validated experimentally.

In this study, we address these questions by learning a continuous representation of voxelated architectures and searching directly in that structural space, rather than restricting optimization to predefined geometric parameters. We represent architected materials as cubic‐symmetric voxel fields (Figure 1A), use a β‐variational autoencoder (β‐VAE) to embed those fields in a continuous latent space [22, 23], and perform multi‐objective optimization directly in that learned representation. A genetic algorithm, here Non‐dominated Sorting Genetic Algorithm II (NSGA‐II), explores this latent space with surrogate models for relative density and relative Young's modulus guiding the search, while an active learning loop [24, 25] repeatedly selects promising candidates at the boundaries of the known design space, evaluates them via high‐fidelity simulations, quantifies their structural novelty retrospectively, and retrains the models in newly explored regions (Figure 1B). This iterative refinement progressively expands the validated design domain, enabling the discovery of architectures that lie beyond the reach of the parameterized families in the initial dataset (Figure 1C). The computationally discovered designs are fabricated via two‐photon polymerization (2PP) and subsequent pyrolysis, and their superior stiffness‐to‐weight ratios are validated through experimental compression tests (Figure 1D,E).

**FIGURE 1:**
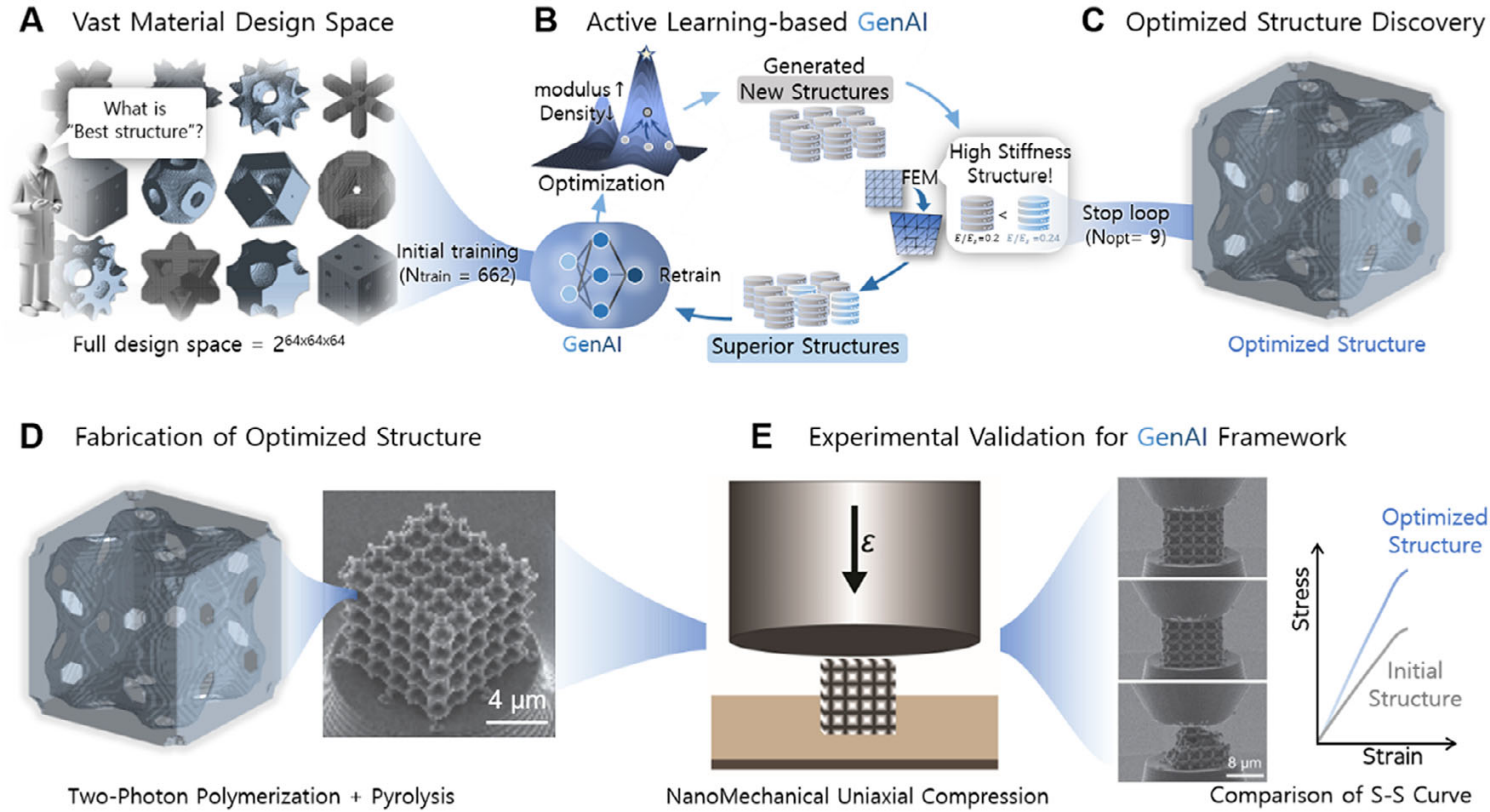
Overview of the generative design and experimental validation framework. (A) A mixed‐family dataset comprising strut‐based, TPMS‐based, and hybrid architectures is assembled as the initial set (N = 3675). (B) A variational autoencoder learns a continuous latent representation from the dataset. Multi‐objective optimization (maximizing modulus and minimizing density) is performed in the latent space, and generated candidate structures are evaluated by finite element simulation. FEM‐validated structures are added to the dataset, and the model is retrained in an active learning loop until the Pareto hypervolume approaches saturation. (C) Representative optimized architecture discovered through the framework. (D) The optimized design is fabricated as a carbon nano‐architecture via two‐photon polymerization and pyrolysis. (E) Quasi‐static uniaxial compression with in situ SEM imaging confirms that the optimized structure outperforms the initial structure in stiffness and strength.

The contribution of the work is threefold. First, we propose a mixed‐family, structure‐centric representation that is not tied to any single analytical topology family. Second, we show that iterative active learning, combined with retrospective novelty quantification, can transform generative optimization from static interpolation into a progressive expansion of the validated design domain. Third, we provide evidence that this strategy yields measurable gains in specific stiffness for low‐density architectures, confirmed both computationally and experimentally, even when benchmarked against the best structures already present in the initial dataset.

## 2 Results

### 2.1 Iterative Discovery of Architected Materials

The optimization framework developed in this study integrates four components into a unified pipeline for structure‐centric discovery of nano‐architectures (Figure 2). A β‐variational autoencoder (β‐VAE) compresses the high‐dimensional voxelated unit cells into a continuous 32‐dimensional latent space (Figure 2A,C). We adopted a β‐VAE because the additional latent regularization helps provide a smoother and more navigable design space for optimization while preserving sufficient reconstruction fidelity. The optimization uses two property predictors with distinct roles: a supervised modulus predictor, ${\bar{E}}_{pred}=P_{M}\left(z_{i}\right)$, and a decoder‐based density predictor, ${\bar{\rho }}_{pred}=P_{D}\left(z_{i}\right)=\phi \left(D_{\theta }\left(z_{i}\right)\right)$, where ***P**_M_*, ***P**_D_*, and ***D***_θ_ are modulus predictor, density predictor, and the β‐VAE decoder, respectively. ϕ computes the solid volume fraction of the reconstructed voxel field. A genetic algorithm (NSGA‐II) then searches the latent space with these two objectives, maximizing relative modulus and minimizing relative density, to identify candidate Pareto‐optimal solutions. In each optimization round, the search yields approximately 100 candidate latent vectors ($z_{i}^{*}$) for downstream decoding and evaluation. The candidate latent vectors are decoded into voxelated unit cells by the β‐VAE decoder, and the reconstructed structures are then evaluated using high‐fidelity finite element simulations to obtain reference mechanical properties. Candidates that return a valid finite element result are incorporated into the training set for the next round.

**FIGURE 2:**
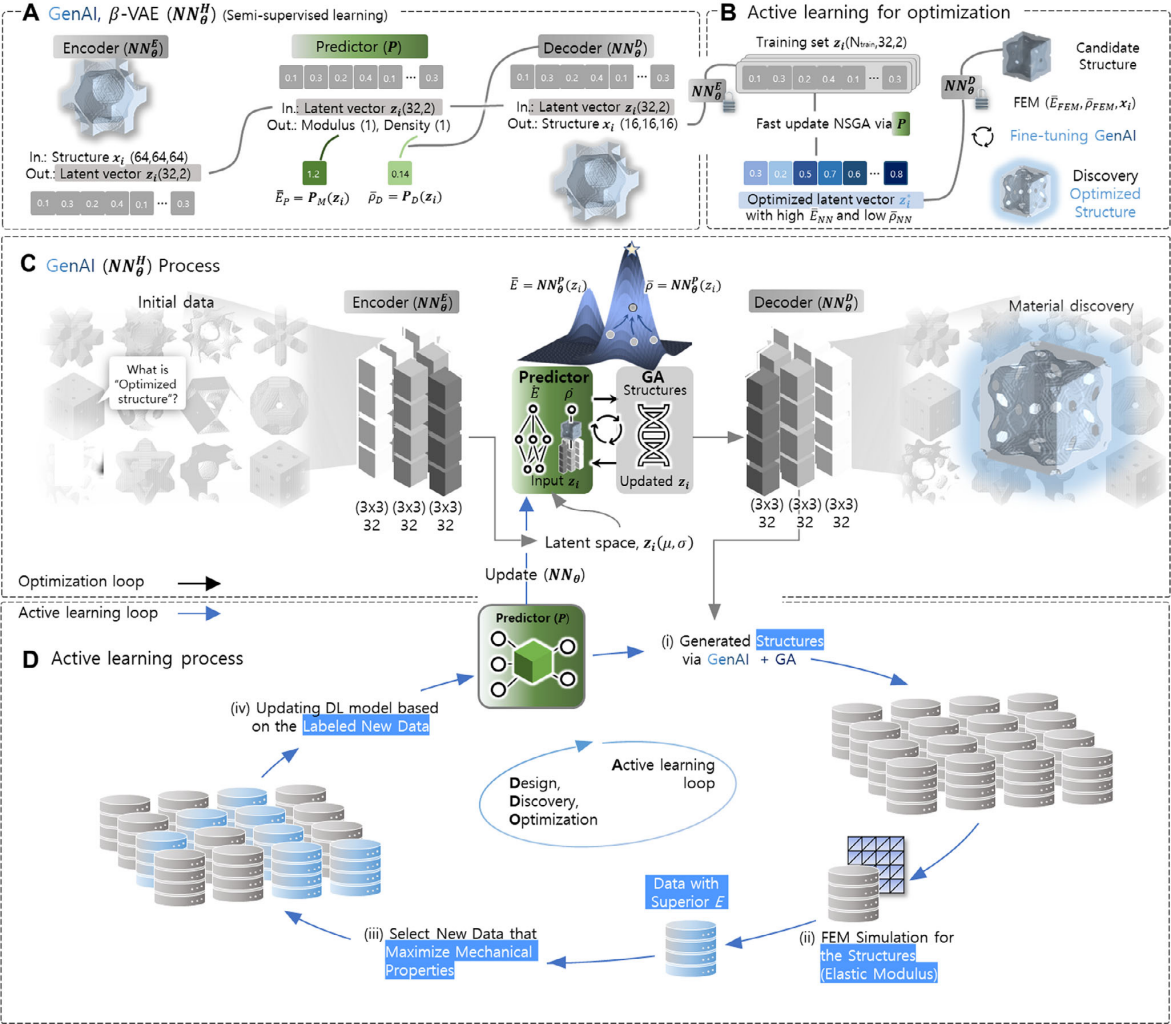
Detailed schematic of the generative optimization framework. (A) The core generative model is a β‐VAE trained in a semi‐supervised fashion. It consists of an encoder, a decoder, and a property surrogate model that learns to map the latent vector directly to modulus. The decoder was used as predictor for density. (B) The active learning optimization strategy combines a fast‐update loop, where a genetic algorithm (GA) is guided by the surrogate model, and a fine‐tuning loop, where novel structures are validated with high‐fidelity FEM simulations. (C) The inner optimization loop in detail. The GA explores the latent space (z), using a surrogate model (ANN) to rapidly predict properties, update the latent vectors, and generate new candidate structures via the decoder. (D) The outer active learning loop. (i) Structures generated from the inner loop are (ii) evaluated via high‐fidelity FEM simulation. (iii) the newly labeled data are collected and (iv) used to retrain all deep learning models (β‐VAE and surrogate model), iteratively improving their accuracy and expanding the explored design space.

Critically, an active learning loop couples latent‐space search to iterative high‐fidelity validation (Figure 2B,D). In each round, promising candidates are selected on the basis of predicted performance, evaluated by finite element simulation, and incorporated into the training set, and their structural distance from the previously validated dataset is quantified retrospectively. Both the β‐VAE and the surrogate are then retrained before the next search round begins. This organization reflects a deliberate design choice. The β‐VAE provides a navigable design space that is not tied to any single topology family. The surrogate makes large‐scale search computationally feasible. NSGA‐II resolves the stiffness–density trade‐off without requiring a single weighted objective. The active learning loop limits unvalidated extrapolation by iteratively converting structurally novel predictions into validated training data. Together, these components form a closed loop for structured exploration rather than one‐shot prediction. The following sections evaluate each stage of this pipeline and present the resulting material discoveries.

### 2.2 Structure‐Centric Representation for Morphology Diversity

Our first objective was to determine whether a mixed‐family voxel representation could serve as a meaningful design basis for high‐specific‐stiffness architected materials. Each candidate structure was discretized on a 64 × 64 × 64 voxel grid. The 64^3^ resolution was verified by a resolution‐sensitivity study (Figure S1). Mesh refinement of the identical geometry up to 256^3^ changes the effective modulus by at most 0.7%, an order of magnitude below the reported design gains, while coarsening to 32^3^ changes it by up to 14% and coarsening further collapses the geometry. To reduce redundant degrees of freedom and limit directional bias during screening, cubic symmetry was imposed so that each structure could be represented by its asymmetric unit. Under the 48 symmetry operations of the cubic group, the full voxel grid was represented by an asymmetric unit (Figure 3A). Boundary voxels shared by multiple symmetry copies lead to 5984 independent grid points, corresponding to an effective numerical reduction of approximately 44‐fold. This reduction is substantial while preserving the broad structural vocabulary rather than collapsing the design into a conventional parametric family.

**FIGURE 3:**
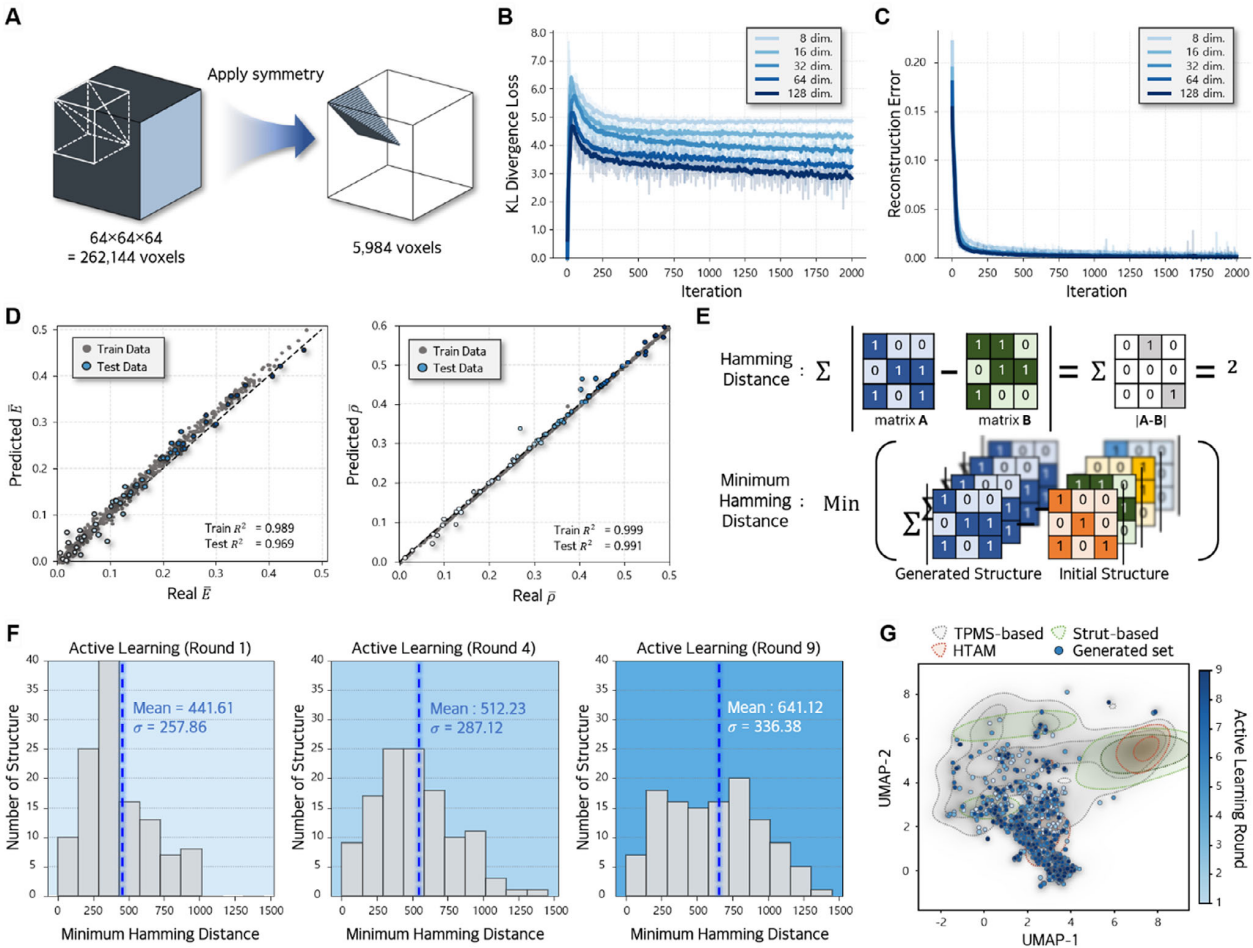
Validation of the ***β***‐VAE, surrogate model, and novelty metric across active learning rounds. (A) Reduction of the computational domain using geometric symmetry. The full **64 × 64 × 64** voxel model (262,144 voxels) is reduced to an irreducible region containing 5,984 voxels. (B, C) Training convergence of the ***β***‐VAE, showing KL divergence (B) and reconstruction error (C) for latent dimensions of 8, 16, 32, 64, and 128. Reconstruction error converges rapidly for all dimensions, while KL divergence shows a clear dependence on dimensionality, stabilizing at lower values for higher dimensions. (D) Parity plots for the surrogate model predictions of relative Young's modulus (left, test ***R*** ^2^ = 0.969) and relative density (right, test ***R*** ^***2***^ = 0.991), confirming high predictive accuracy on held‐out test data. (E) Schematic illustration of the novelty metric. The Hamming distance (top) counts the bitwise differences between two binary voxel fields. The minimum Hamming distance (MHD, bottom) is defined as the smallest Hamming distance from a candidate structure to any structure in the existing validated dataset. (F) Histograms of the minimum Hamming distance (MHD) for candidates validated in active learning rounds 1, 4, and 9. The mean MHD increased from 441.61 to 641.12, and the standard deviation, ***σ***, increased from 257.86 to 336.38, indicating that later rounds validated structures farther from the existing dataset with a broader spread of structural distances. (G) UMAP embedding of the learned latent space. Grey shading, embedding density of the initial dataset; contours, 50% and 90% density levels of each source family (TPMS‐based, strut‐based, HTAM); circles, FEM‐validated candidates colored by active learning round.

This choice of representation matters because the learning problem is fundamentally different from family‐specific optimization. When a TPMS level‐set offset, a truss radius, or a porosity field is optimized, the search operates inside a geometry class that is already known. In contrast, a voxelated representation allows the learning algorithm to encode local and nonlocal geometric correlations without presupposing whether the efficient design should resemble a shell, a truss, a hybrid, or something entirely distinct. The mixed‐family dataset was assembled with this purpose in mind. It included 152 strut‐based lattices (both stretching‐ and bending‐dominated), 3420 TPMS‐based architectures (shell and volumetric), and 103 hybrid TPMS‐based architected materials (HTAM) so that the latent space would be exposed to multiple mechanically relevant modes of material organization rather than one privileged topology (Figure S2). The family counts were not intended to be numerically balanced. Instead, they reflect differences in accessible parameterization. Strut‐based lattices were varied primarily through member thickness over a limited set of base topologies, whereas TPMS‐based architectures could be sampled more densely through independent variation of thickness and level‐set offset. The larger number of TPMS‐based structures should therefore be understood as a consequence of their richer parametric sampling space, rather than as an intentional preference for a single architecture class.

The dataset was further curated to emphasize the low‐density regime and remove mechanically uninformative designs. The density cutoff ($\bar{\rho }<0.6$) removes near‐solid structures that are less relevant to lightweight design, whereas the stiffness‐scaling criterion ($\bar{E}>{\bar{\rho }}^{2}$) excludes mechanically inefficient architectures such as disconnected fragments or poorly load‐bearing morphologies. This threshold was chosen to remove designs whose stiffness fell substantially below the scaling expected of mechanically efficient cellular architectures in the targeted density regime [26]. This filtering step is important scientifically, not merely computationally. In a broad, unfiltered dataset, a generative model can devote excessive capacity to reproducing trivial or obviously suboptimal structures, such as near‐solid blocks or disconnected fragments. By concentrating the training set on architectures that are both lightweight and mechanically meaningful, the model is encouraged to learn distinctions that matter near the stiffness‐density frontier. The curation therefore reflects the intended discovery problem. Rather than reconstructing all possible shapes, we aim to navigate the subset of structural space in which improvements in geometric efficiency are most consequential. The value of this mixture is visible before any search. The Pareto front of the TPMS members alone has a hypervolume of 0.098 against 0.106 for the mixed set, most of the difference being contributed by the 37 HTAM members retained after filtering, and a search started from the TPMS members alone does not recover that frontier within nine rounds (Figure S3).

Because the learning objective is ultimately mechanical rather than geometric, all properties were evaluated in normalized form. Relative density ($\bar{\rho }=\rho /\rho_{s}$, where ρ is the density of the architected material and ρ_*s*_ is the density of the solid constituent) and relative Young's modulus ($\bar{E}=E_{eff}/E_{s}$, where *E_eff_* and *E_s_* are effective modulus of architected material and modulus of solid, respectively) were defined with respect to the properties of the solid constituent material, thereby isolating the effect of architecture from the choice of base material. Effective stiffness was obtained by linear elastic finite‐element‐based numerical homogenization of the voxelated unit cells. This normalization ensures that improvements attributed to the generative framework arise from better material distribution and load transfer rather than from changes in constituent chemistry or absolute scale.

We next asked whether this high‐dimensional representation could be compressed without losing the structural information required for property prediction and optimization. VAEs with latent dimensions of 8, 16, 32, 64, and 128 were therefore trained and evaluated using both reconstruction error and Kullback–Leibler (KL) divergence (Figure 3B,C). Reconstruction error remained low across the tested range, indicating that the cubic‐symmetric voxel fields are highly compressible. The KL divergence decreased from approximately 4.9 and 4.2 for 8‐ and 16‐dimensional models to 3.7, 3.2, and 2.9 for 32‐, 64‐, and 128‐dimensional models, respectively, while the reconstruction error remained below 10^−3^ in all cases. Because evolutionary search becomes increasingly inefficient in high‐dimensional decision spaces, we selected 32 latent dimensions as a practical compromise between reconstruction fidelity, latent regularity, and NSGA‐II search tractability. This choice provided sufficient compactness, distributional regularity, and downstream optimization efficiency.

### 2.3 Latent‐Space Fidelity and Predictor Accuracy

A useful latent representation must do more than reconstruct training examples; it must also support the prediction of properties that matter for design. As defined above, the optimization used two property predictors with distinct roles: a supervised modulus predictor and a decoder‐based density predictor. On a held‐out random test split, the decoder‐based density predictor achieved an *R* ^2^ of 0.991, while the supervised modulus predictor achieved an *R* ^2^ of 0.969 (Figure 3D). The higher accuracy for density is expected because density is directly related to voxel occupancy, whereas modulus emerges from a more global interplay of connectivity, slenderness, curvature, and load path.

These predictors make the overall optimization problem computationally feasible. Directly coupling a population‐based optimizer to high‐fidelity finite element simulations for every candidate in every generation would be prohibitively expensive. By replacing most modulus evaluations with supervised predictions and estimating density from the decoded voxel occupancy, the framework can explore large numbers of candidates while reserving full finite‐element validation for strategically selected designs.

The multi‐objective search itself was carried out using NSGA‐II, with relative density minimized and relative Young's modulus maximized. This choice reflects the fact that architected materials are fundamentally trade‐off systems. Because NSGA‐II preserves diversity through non‐dominated sorting and crowding distance, it is well suited to problems where multiple high‐performing design families may coexist [27]. That feature is especially valuable in a mixed‐family latent space, where the most informative outcome is not necessarily one best point but a frontier containing distinct architected material morphologies. In this way, predictor‐guided latent search enables the algorithm to propose mechanically plausible candidate structures that are not restricted to the parameterization of any single topology family.

### 2.4 Design Domain Expansion and Pareto‐Front Propagation by Active Learning

A central challenge in generative optimization is to determine whether iterative search expands the validated design domain rather than merely densifying sampling around known structures. This distinction motivated our analysis of structural novelty during the active learning process. After each round of predictor‐guided latent search, selected candidates were decoded, evaluated by high‐fidelity finite element simulation, and added to the training set for model retraining. Candidate selection was based on predicted mechanical performance, whereas structural novelty was assessed retrospectively to evaluate whether the active learning process progressively moved into regions not well represented by the existing validated dataset.

To quantify structural novelty, we used the minimum Hamming distance (MHD) between each generated structure and the existing validated dataset (Figure 3E). Each voxelated candidate was compared with all previously validated structures, and the smallest binary difference was taken as its novelty score. A structure is therefore treated as novel only when it is distant from its nearest known neighbor, rather than merely different from an average design or a chosen prototype. MHD is well matched to the question posed here: whether newly validated architectures are moving outward from the region already covered by the data.

Across successive active learning rounds, the mean MHD increased from 441.61 (7.4% of the 5984 independent voxels) in the first round to 641.12 (10.7%) in the ninth round, while the standard deviation increased from 257.86 to 336.38 (Figure 3F). The increase in mean MHD indicates that newly validated structures became progressively farther from the existing dataset, whereas the larger standard deviation suggests a broader absolute spread of structural distances in later rounds. This systematic growth indicates that retraining did not merely densify sampling inside the initial cloud of structures. Instead, the active learning process progressively validated candidates farther from previously validated structures. MHD is a dataset‐relative measure and does not establish global novelty across all possible architected materials. Nevertheless, its increase supports the conclusion that the workflow expands the validated design domain relative to the initial dataset.

To complement this voxel‐space novelty analysis, we visualized the learned latent space using Uniform Manifold Approximation and Projection (UMAP), which projects high‐dimensional latent vectors into a 2D embedding while preserving local neighborhood relationships (Figure 3G). The UMAP map shows that the active learning candidates do not scatter randomly across the latent space, but instead occupy a coherent region that was sparsely populated by the initial design set. Resolved by source family, the candidates lie outside the 50% density cores of the TPMS‐based, strut‐based, and HTAM members alike. Quantitatively, the mean local embedding density of the initial data at the active learning candidate locations was approximately 49% of the initial‐data peak density, and 91% of the candidates were located in regions below the median initial‐data embedding density. Although UMAP is a nonlinear visualization and should not be interpreted as a direct metric of global latent‐space distance, this analysis supports the MHD result by showing that active learning moved the search toward under‐sampled but coherent regions of the learned design space.

### 2.5 Frontier Expansion in Low Density, High Stiffness Domain

The next test of the framework is whether active learning identifies mechanically improved designs rather than merely structurally different ones. In the stiffness–density map, the optimized structures advance the Pareto frontier toward the linear‐scaling reference $\bar{E}=\bar{\rho }$, corresponding to the Voigt upper benchmark for stiffness at a given volume fraction [28], and approach it more closely than the initial designs at comparable density (Figure 4A).

**FIGURE 4:**
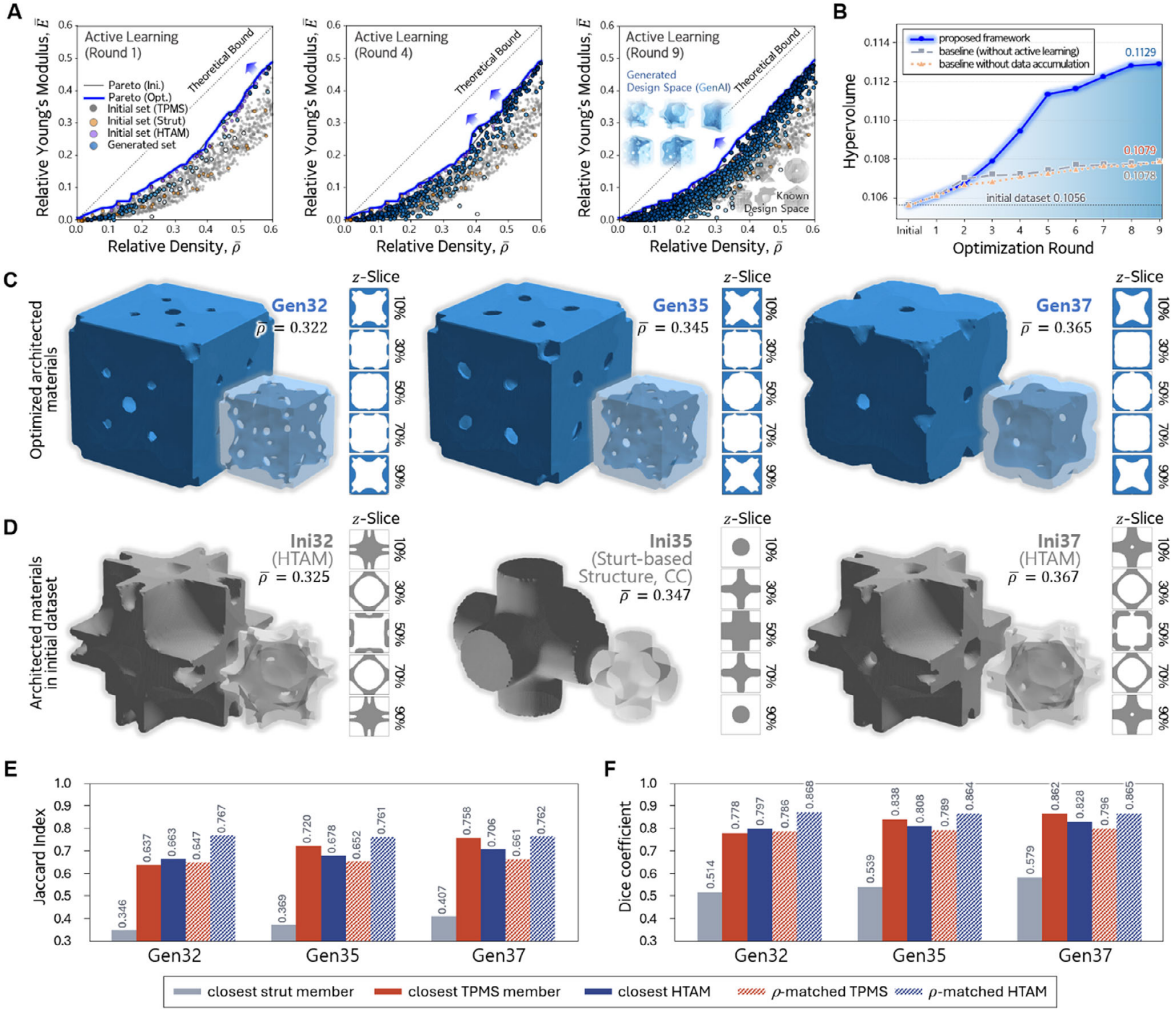
Pareto‐front progression, hypervolume evolution, and density‐matched architecture comparison. (A) Stiffness–density maps at active learning rounds 1, 4, and 9. Initial dataset colored by family (TPMS‐based, grey; strut‐based, orange; HTAM, purple); blue circles, generated structures; thin line, Pareto front of the initial dataset; blue line, cumulative Pareto front; dotted line, linear‐scaling reference $\left(\bar{E}=\bar{\rho }\right)$. Insets, representative structures generated in round 9. (B) Pareto hypervolume of the cumulative Pareto front per round for the proposed framework, the baseline without active learning, and the baseline without data accumulation. Dotted line, hypervolume of the initial training dataset. (C) Optimized architectures selected from the final Pareto front, Gen32, Gen35, and Gen37, with representative ***z***‐slices shown below each structure. Their relative densities are 0.322, 0.345, and 0.365, respectively. (D) Best‐performing initial‐dataset counterparts, Ini32, Ini35, and Ini37, with representative ***z***‐slices shown below each structure. Their relative densities are 0.325, 0.347, and 0.367, respectively. (E) Jaccard index and (F) Dice coefficient between each optimized design and the closest strut‐based, TPMS‐based, and HTAM members of the initial dataset (solid bars) and the density‐matched TPMS and HTAM parameterization fits (hatched bars).

The Pareto front advanced first in the relatively higher‐density region (round 1) and only later in the lower‐density regime (round 9) (Figure 4A). This progression suggests that the model first learned mechanically effective motifs from stronger moderate‐density structures ($\bar{\rho }>0.5$) in the initial dataset and then extended those motifs toward lower‐density designs through active learning. This shift is particularly important because low‐density structures are inherently more difficult to improve. Reduced material fraction leads to weaker connectivity and makes stiffness highly sensitive to inefficient load paths, local stress concentration, and poorly distributed curvature. As a result, improvements in the low‐density regime cannot be explained simply by increasing material fraction but instead reflect more efficient material distribution at comparable density.

Objective‐space evidence supports the same interpretation. Pareto hypervolume quantifies the objective‐space region dominated by the Pareto front, so an increase indicates simultaneous improvement in the stiffness–density trade‐off. The Pareto hypervolume, evaluated with respect to the reference point (relative density 0.6, relative modulus 0), increased from 0.106 for the initial training dataset to 0.113 after nine active learning rounds, with most of the gain achieved by approximately the fifth round and subsequent changes approaching saturation (Figure 4B). To attribute the improvement, we repeated the optimization with the generative model and surrogate kept fixed at their initial state while keeping the search algorithm, seeding, density window, selection rule, and FEM budget identical (100 candidates per round, nine rounds). Because the first round precedes any retraining, both arms share its candidates and diverge only from round 2. The baseline then advanced only slowly over eight rounds (hypervolume 0.1061 to 0.1078, against 0.1129 for the active learning loop, a gain three to four times larger). In the density band of the fabricated designs (relative density 0.30 to 0.40) it never exceeded the 0.213 inherited from the first round, against 0.262 for the active learning loop (Figure S4). Under a matched measurement protocol, in which each round's candidates are scored by the surrogate that proposed them, that is, the model trained on the data available before that round (the fixed round‐1 model throughout for the baseline), both surrogates remained well calibrated on their own acquired candidates (*R* ^2^ = 0.91–0.96 baseline vs 0.89–0.97 retrained), so the baseline search stalls by confinement to the already‐covered region rather than by miscalibration (Figure S5).

Hypervolume alone does not diagnose structural novelty, because a frontier can move without changing the underlying nature of the designs. However, considered together with the increasing MHD, it indicates that the workflow improved the stiffness–density frontier while also expanding the validated design domain relative to the initial dataset.

We therefore compared optimized structures with the best‐performing members of the initial dataset at nearly matched relative densities. For clarity, optimized and initial structures are labeled by their approximate relative density. For example, Gen32 denotes an optimized architecture at $\bar{\rho }\approx 0.322$, whereas Ini32 denotes its nearest initial‐dataset counterpart at $\bar{\rho }\approx 0.325$. The selected optimized architectures, Gen32, Gen35, and Gen37, and their corresponding initial counterparts, Ini32, Ini35, and Ini37, are shown with representative *z*‐slices in Figure 4C,D. These slice views highlight the internal structural differences between the optimized and initial designs at comparable density. The optimized architectures also show more gradual geometric variation along the *z*‐direction, whereas the initial counterparts contain sharper transitions in local geometry and connectivity.

To confirm that these differences constitute structural novelty rather than a variation of existing designs, we quantified the geometric overlap between each optimized architecture and its closest counterparts (Figure 4E,F). The Jaccard index is the intersection of two solid voxel sets divided by their union, and the Dice coefficient is a monotone transform of the same overlap; both equal unity only for identical geometries. Against the most similar member of each family in the initial dataset, the Jaccard index reaches only 0.35 to 0.41 for strut‐based, 0.66 to 0.71 for HTAM, and 0.64 to 0.76 for TPMS‐based structures (Figure 4E). Because a design can be distant from every sampled member yet still lie on a parameterization manifold, we additionally fitted the TPMS and hybrid parameterizations directly, re‐optimized at the density of each design; even these density‐matched best fits saturate at Jaccard indices of 0.65 to 0.66 (single TPMS family) and 0.76 to 0.77 (HTAM parameterization), with Dice coefficients of at most 0.87 (Figure 4F). No tuning route reproduces more than about three quarters of the solid volume of the optimized designs, indicating that they lie outside the sampled neighborhoods of the initial dataset and beyond the reach of its parameterizations. Additionally, by direct FEM even the best density‐matched fits remain less stiff than the generated designs, by 13% to 19% for the single family (relative modulus 0.160, 0.180, and 0.195 against 0.185, 0.207, and 0.241) and by 6% to 17% for the HTAM fits (0.174, 0.171, and 0.209) (Figure S6).

The corresponding relative Young's modulus values calculated by finite element analysis are summarized in Figure 5A, which benchmarks each optimized design against the stiffness distribution of the entire initial dataset (N = 3675) within progressively wider relative‐density windows (±0.005, ±0.01, and ±0.02). Ini32, Ini35, and Ini37 were not hand‐selected. They are the stiffest members of the dataset within ±0.01 relative density of each design. Gen32, Gen35, and Gen37 achieved relative moduli of 0.185, 0.207, and 0.241, whereas Ini32, Ini35, and Ini37 at slightly higher relative densities achieved lower values of 0.166, 0.180, and 0.200. These comparisons correspond to stiffness gains of approximately 11%, 15%, and 20%, despite the optimized structures being marginally lighter. The advantage persists as the comparison broadens. At ±0.02, where each band holds 122 to 140 structures, the gains remain +10.5%, +6.5%, and +16.1%. Even against every initial structure no denser than each design, which removes the built‐in advantage of denser members, the optimized designs remain 20.7%, 23.9%, and 24.2% stiffer (Table S1). Because stiffness is strongly coupled to density in cellular solids, these density‐matched gains support an improvement in geometric material distribution efficiency rather than a benefit from increased material fraction.

**FIGURE 5:**
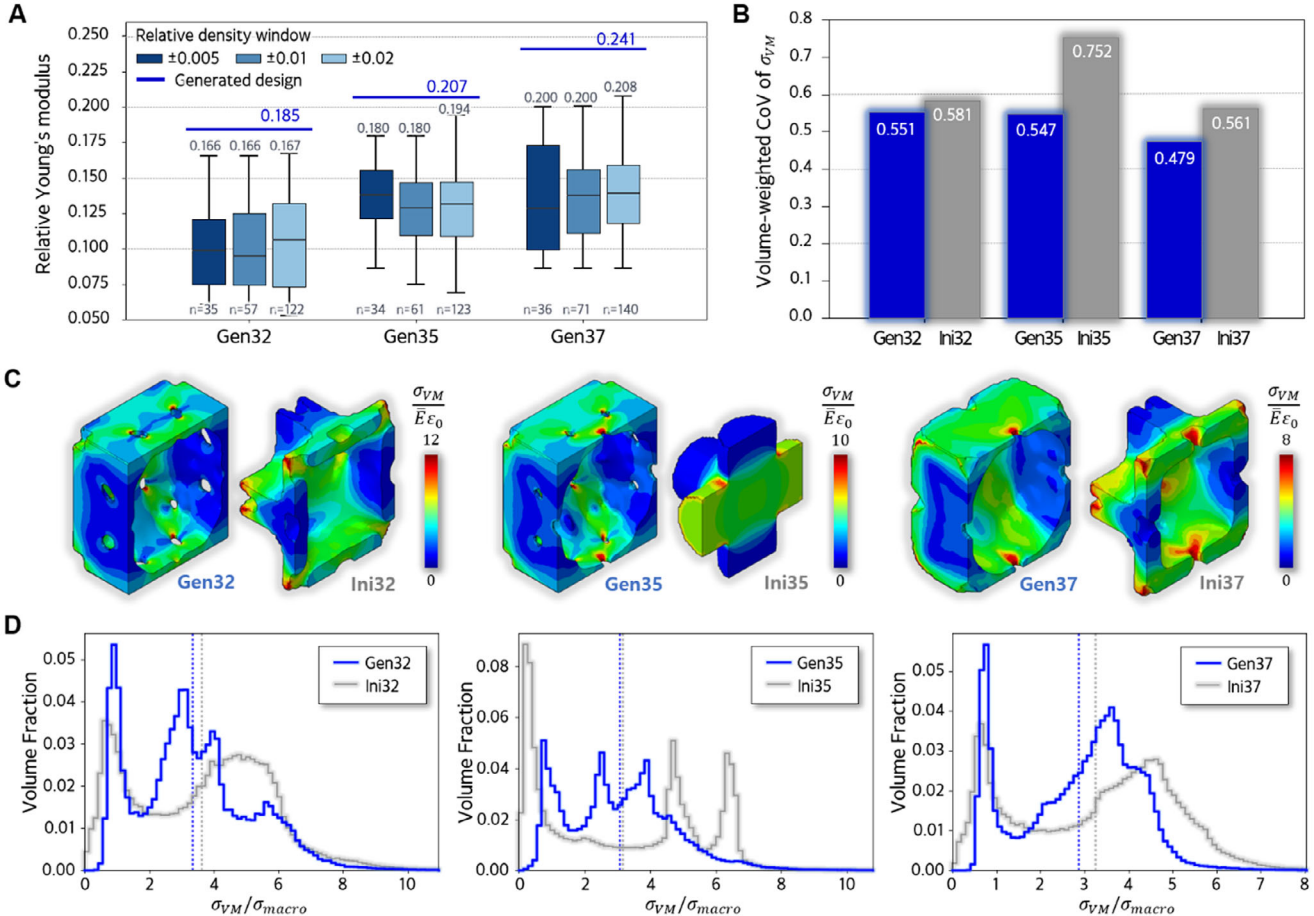
Stiffness comparison at matched density and stress‐field analysis of optimized and initial architectures. (A) Relative Young's modulus of all members of the initial dataset (N = 3675) within ±0.005, ±0.01, and ±0.02 relative density of each optimized design (box, interquartile range; whiskers, full range; n, number of members). Horizontal lines, optimized designs. (B) Volume‐weighted coefficient of variation (CoV) of normalized von Mises stress for each architecture. Lower CoV indicates a more spatially uniform stress distribution within the solid phase. (C) Normalized von Mises stress fields for Gen32, Ini32, Gen35, Ini35, Gen37, and Ini37. The local von Mises stress is normalized by the macroscopic stress, ***σ***_**macro**_ = ***E***_**eff**_ ***ε***_0_, as ${\hat{\sigma }}_{\mathrm{VM}}=\sigma_{\mathrm{VM}}/\sigma_{\mathrm{macro}}$. (D) Volume‐fraction histograms of normalized von Mises stress. The optimized architectures show a larger fraction of material at lower normalized stress and reduced high‐stress localization relative to the corresponding initial structures.

To examine the mechanical origin of these density‐matched stiffness gains, we compared the local stress distributions of the optimized and initial architectures under the same macroscopic loading condition. The local von Mises stress was normalized by the macroscopic stress, σ_macro_ = *E*_eff_ε_0_, giving ${\hat{\sigma }}_{\mathrm{VM}}=\sigma_{\mathrm{VM}}/\sigma_{\mathrm{macro}}$. This normalization enables stress localization to be compared across architectures with different effective stiffness values. We then evaluated the volume‐weighted coefficient of variation of ${\hat{\sigma }}_{\mathrm{VM}}$, where a lower value indicates a more spatially uniform stress distribution within the solid phase. The optimized architectures exhibited lower stress heterogeneity than their initial counterparts, with CoV values of 0.551 versus 0.581 for Gen32/Ini32, 0.547 versus 0.752 for Gen35/Ini35, and 0.479 versus 0.561 for Gen37/Ini37 (Figure 5B). The normalized stress fields and volume‐fraction histograms show the same trend (Figure 5C,D). Compared with the initial designs, the optimized architectures contain a larger fraction of material at lower normalized stress and show reduced high‐stress localization. The contrast is particularly pronounced for Ini35, whose strut‐based geometry produces localized stress concentrations near nodal regions. These results support the interpretation that the optimized architectures improve stiffness by distributing load more effectively at comparable density.

This comparison also clarifies the value of structure‐centric search. The initial dataset already contained strong designs drawn from advanced architecture families, including hybrid TPMS‐based structures. The optimized candidates therefore outperform not a weak or naive reference set, but a curated set of already competitive structures. This makes the observed gains more meaningful. If the starting point had been an unsophisticated design library, the improvement could have been attributed primarily to baseline selection. Here, the competitive reference set suggests that the remaining gains arise from searching beyond the local parameterization of the initial designs.

### 2.6 Nanomechanics of Optimized Architectures

Experimentally validating the mechanical enhancements by the optimization process, the three selected generated architectures (Gen32, Gen35, Gen37) and associated three initial architectures (Ini32, Ini35, Ini37) were fabricated as carbon nanoarchitectures using two‐photon polymerization followed by carbothermal decomposition. The three optimized Gen architectures are shown in Figure 6A‐C (Ini structures in Figure S7). X‐ray photoelectron spectroscopy (XPS) and Raman spectroscopy show signatures of approximately 90% carbon with typical few‐nm aromatic carbon fragments as commonly noted in two photon polymerization‐derived pyrolyzed carbon (Figure S8). Internal geometry was examined by cross‐sectional focused ion beam (FIB) cutting with scanning electron microscope (SEM) imaging (Figure S10). Individual wall and strut thicknesses are on the order of 220 to 400 nm, and the designed morphology is preserved, with local deviations in wall thickness and pore width at the level of the measurement increment. These 2D cross sections do not determine the 3D solid volume fraction, which is taken from the CAD model throughout.

**FIGURE 6:**
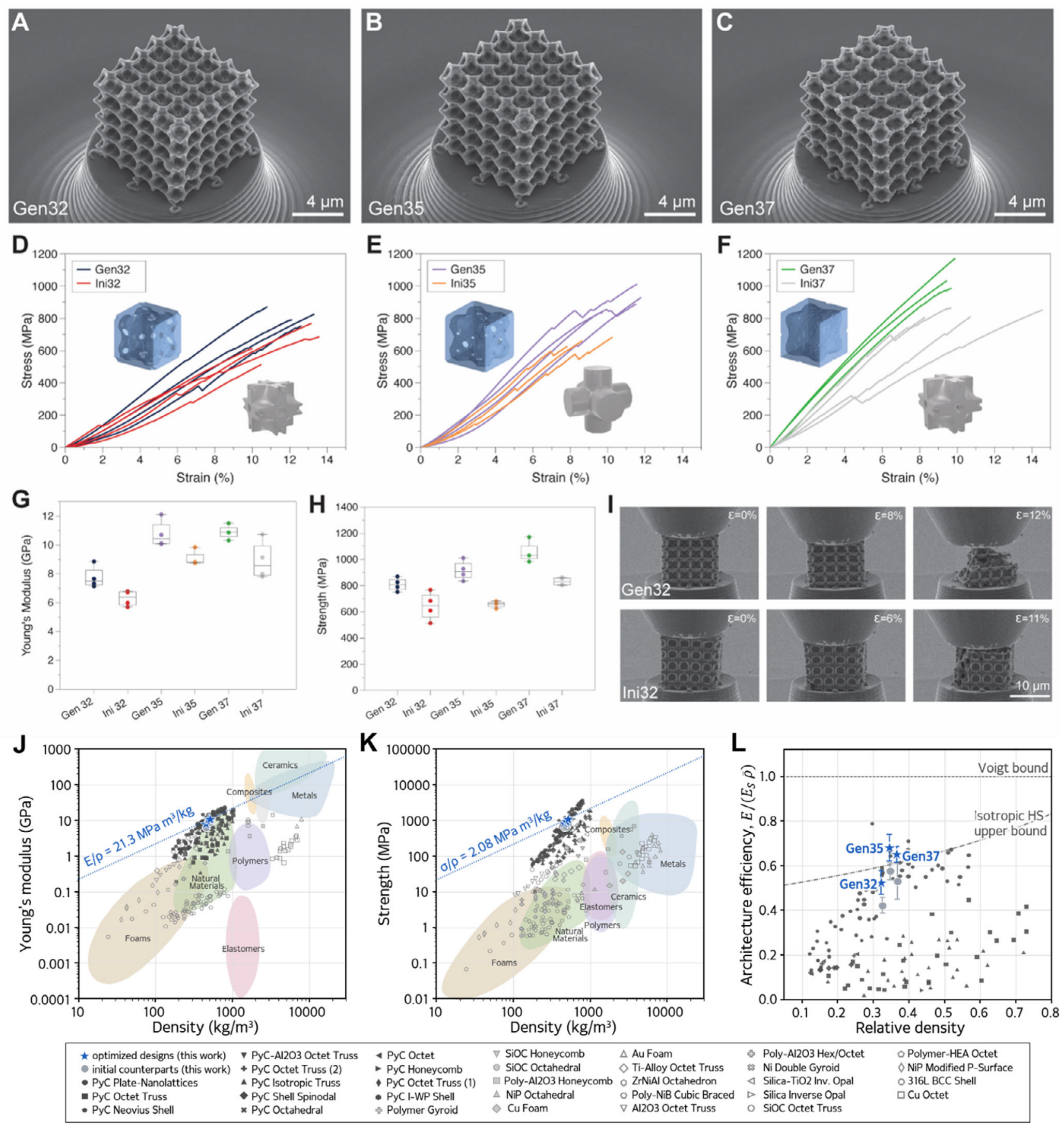
Nanomechanical evaluation of generative geometries. (A‐C) Carbon nanolattices derived from two photon polymerization and carbothermal reduction pyrolysis of generative designs at 32%, 34.5%, and 36.5% relative density. (D‐F) Nanomechanical uniaxial compression quasi‐static stress‐strain curves of generative and initial nanoarchitectures across three relative densities. (G, H) Young's modulus and strength summary of uniaxial compression tests. (I) In situ SEM images of deformation mechanisms during compression for 32% relative density generative and initial structures. (J) Young's modulus and (K) strength against density for the optimized designs, their initial counterparts, nanoarchitected materials from the literature. (L) Architecture efficiency $E/(E_{s}\bar{\rho })$ against relative density for the pyrolytic‐carbon lattices, normalized by the fitted constituent modulus (Experimental Section). Error bars, one standard deviation. Sources of the literature data, Table S2.

The nanoarchitectures are subjected to quasi‐static uniaxial compression with SEM in situ imaging. All three generative structures show enhanced Young's moduli and strength as compared to their respective initial structures with slightly higher relative densities (Figure 6D‐F). The failure strain is consistent between Gen32, Gen35, Gen37, Ini32 and Ini37 cases, while the Ini35 structure shows reduced failure strain by more than 2% compared to the Gen35. This is likely due to the stress concentrations occurring at the node for the strut‐based geometry, as opposed to the other five geometries, which are smooth, volumetric architectures. The summarized Young's modulus and strength are presented in Figure 6G,H. The Young's moduli are 7.75 ± 0.77, 10.76 ± 0.95, and 10.90 ± 0.60 GPa for Gen32, Gen35, and Gen37, against 6.30 ± 0.54, 9.14 ± 0.62, and 8.92 ± 1.35 GPa for Ini32, Ini35, and Ini37, and the strengths are 809 ± 50, 916 ± 75, and 1062 ± 98 MPa against 644 ± 108, 656 ± 29, and 832 ± 30 MPa. The Young's moduli are therefore increased by 23%, 18%, and 22% and the strength by 26%, 40%, and 28% (Welch's *t*‐test, p = 0.025, 0.042, and 0.055 for the modulus and p = 0.047, 0.003, and 0.047 for the strength). These experimental gains are comparable in magnitude to the improvements predicted computationally, providing validation of the optimization framework. The notably larger strength gain for Gen35 likely reflects the poor stress distribution at the nodes of its strut‐based counterpart Ini35, as compared to the even stress distribution of the smooth, volumetric architectures. Although the optimization targeted only the linear‐elastic stiffness, the fabricated designs also showed higher strength. This is consistent with the lower stress heterogeneity of the optimized architectures in simulation and with the distributed deformation observed in situ, since a more uniform load distribution delays the local failure that sets the strength of a brittle constituent. The linear‐elastic simulations do not model crack initiation or propagation, so they do not predict strength directly.

In situ SEM images during uniaxial compression show notable differences in deformation and failure modes for the generative structures as compared to the initial structures. Figure 6I shows the Gen32 and Ini32 subject to compression to failure (other nanoarchitectures in Figure S9). The generative design shows global deformation without any visible damage up to ε = 8%, followed by global cracking and failure. Conversely, the initial structure shows global deformation with evidence of damage at ε = 6% followed by erratic crack propagation at ε = 11%. The increase in global deformation without damage followed by widespread failure is indicative of distributed loading afforded by the generative design as compared to the initial structure. While many architected designs exhibit layer‐by‐layer failure due to stress accumulation in the contact region [29], the generative designs effectively distribute stress throughout the wider volume, leading to enhanced Young's moduli and strength. This agrees with the reduced stress heterogeneity and CoV of the generative structures as per Figure 5B‐D, enabling global deformation without premature failure at concentrated stress points. The novel geometries predicted by this generative framework have fine details of few‐voxel resolution and unconventional curvatures that enable these theoretical enhancements. The nanoscale resolution of two photon polymerization and pyrolysis enables sufficiently high manufacturing fidelity to capture these key geometric features, effectively translating these theoretical stress distributions into physical deformation modes.

At *E*/ρ = 17.2, 22.3, and 21.3 MPa m^3^ kg^−1^ and σ/ρ = 1.79, 1.90, and 2.08 MPa m^3^ kg^−1^ for Gen32, Gen35, and Gen37, respectively, the three generative geometries lie in the upper range of nanoarchitected materials (Figure 6J,K). At comparable density they exceed the truss‐based pyrolytic carbon, metallic, and polymer‐derived nanolattices reported to date, match the stiffest ceramic octet trusses while exceeding them in strength [1, 4, 6, 29, 30], and fall within the cluster of the shell‐ and plate‐based pyrolytic carbon nanolattices that lie closest to the theoretical bounds [3, 31], whose stiffest members exceed them by up to about 60% in stiffness. Normalized by the constituent modulus, the generative geometries reach an architecture efficiency comparable to that of these plate‐ and shell‐based designs, and Gen35 and Gen37 exceed most of them (Figure 6L).

## 3 Discussion

The results of this study support a broader principle for architected material design. Optimization outcomes depend not only on the search algorithm or predictor fidelity, but also on what the underlying representation makes searchable. By using a VAE to compress high‐dimensional voxelated unit cells into a continuous latent space, the framework makes direct exploration of voxel‐based structures computationally tractable while retaining sufficient morphological information for design. This representation enables the search to move across a broader morphology space than any parameter‐bound topology family spans, allowing the framework to propose structures that are not prescribed by a single analytical form. FEM‐based validation through active learning further grounds this expanded search in mechanically evaluated structure–property data rather than relying solely on latent‐space predictions.

The experimental compression tests provide independent evidence that the computationally identified gains translate into improved mechanical performance. The optimized structures showed Young's modulus enhancements of 18%–23% and strength enhancements of 26–40% relative to their density‐matched initial counterparts. These gains are comparable to the computationally predicted improvements, indicating that the latent‐space optimization captured mechanically meaningful structural differences rather than only numerical artifacts. The strength gains, although not targeted by the optimizer, are consistent with the stress‐field analysis, which associates the stiffness gain with a more distributed stress state, and with the global deformation observed in situ before distributed cracking. It is notable that the optimized designs converged toward smooth, volumetric morphologies in the $\bar{\rho }$ = 32–37% regime, suggesting that the search favored continuous load‐bearing architectures at these densities.

These findings carry implications beyond the specific carbon nano‐architecture system studied here. The key design variable was not simply whether a structure belonged to a truss, TPMS, or hybrid family, but how effectively material was distributed along load‐bearing pathways within a broader morphology space. This insight is difficult to obtain when optimization is confined to a narrow parameterization, because the search is restricted to structural alternatives already encoded by that parameterization. The framework therefore offers a strategy for problems where the design space is large, heterogeneous, and not fully captured by existing topology families.

The generated architectures are not new topology families in the geometric sense. An inverse fit of the continuous level‐set parameterizations reproduces roughly two thirds of their solid volume. Their distinction is mechanical: at matched density, even the geometrically closest representable structures fall 13% to 19% short in stiffness by direct FEM (Figure S6), so the gains arise from voxel‐scale features that the analytic parameterizations do not capture. Density‐matched fits of the HTAM parameterization narrow but do not close the gap, reaching a Jaccard index of only 0.77 and remaining 6% to 17% short in stiffness by the same FEM test.

The study has several limitations that define the boundaries of the current claims. The validated design space remains conditioned by the initial dataset, and although active learning expands that space, the search cannot be entirely independent of the architectural priors embedded in the seed data. A search started from the TPMS members alone does not recover the frontier of the mixed dataset (Figure S3). Cubic symmetry was imposed to reduce dimensionality and limit directional bias, but this choice excludes intentionally anisotropic architectures that may be optimal for directional loading. The minimum Hamming distance provides a useful but dataset‐relative measure of structural novelty, rather than a universal measure across all conceivable architected materials. The voxel representation also introduces resolution‐dependent discretization that may blur sharp geometric features. Finally, the computational screening was limited to linear‐elastic homogenization, which does not capture nonlinear, post‐yield, buckling, or failure behavior during optimization.

Several extensions would strengthen the framework and broaden its applicability. First, incorporating uncertainty‐aware, novelty‐aware, or multi‐fidelity acquisition strategies could improve candidate selection efficiency, particularly when high‐fidelity simulations are expensive. Second, the objective space should be expanded beyond elastic stiffness and density to include strength, buckling resistance, energy absorption, and multifunctional couplings. Third, richer geometric representations, such as signed‐distance fields, implicit neural surfaces, or graph‐based encodings, may preserve fine structural features more faithfully than binary voxels at a fixed resolution. Fourth, fabrication constraints and process‐induced defect models should be incorporated directly into the optimization loop rather than treated only during post hoc validation. These extensions would move the framework from a proof of concept toward a practical design tool for nano‐architected materials.

## 4 Conclusions

This work asked whether mechanically superior architectures exist beyond the reach of the known parameterized families and whether they can be discovered systematically. The framework answers the discovery question affirmatively in a precise sense. The architectures it finds are geometrically related to the known families yet are not reproduced by density‐matched tuning of them, outperforming the best density‐matched members of the entire initial dataset and of the fitted parameterizations themselves, and a baseline without active learning at identical FEM budget attributes this capability to representation retraining. Across nine active learning rounds, the validated design domain expanded progressively, with the mean minimum Hamming distance from the initial dataset increasing from 442 to 641 and the Pareto hypervolume rising by 7%, and the discovered architectures achieved 11%–20% higher relative Young's modulus than the best initial designs at comparable densities. Fabricated as carbon nano‐architectures, the optimized designs further delivered 18–23% higher Young's modulus and 26–40% higher strength than density‐matched counterparts, reaching specific stiffnesses of 17.2, 22.3, and 21.3 MPa m^3^ kg^−^^1^ in the low‐density regime.

Taken together, the computational and experimental results demonstrate that direct learning in structural space, combined with active learning and retrospective novelty analysis, enables the discovery of mechanically efficient nano‐architectures that are beyond the local neighborhoods represented in the initial design set. More broadly, the results suggest that representation choice, search strategy, and validation procedure jointly shape what kinds of morphologies can be identified in data‐driven architected‐material discovery.

## 5 Experimental Section

### 5.1 Dataset Construction and Design‐Space Curation

The initial dataset comprised 3675 unit‐cell architectures spanning three mechanically distinct classes. The 152 strut‐based lattice structures included CC, BCC, FCC, octet, CBCC, and CFCC topologies. The 3,420 TPMS‐based architected materials were generated from 18 implicit surface families (Schwarz P, Schwarz D, gyroid, Neovius, I‐WP, F‐RD, double diamond, double primitive, C(D), D′, G′, double gyroid, Karcher K, O,C‐TO, Lidinoid, and the Fischer–Koch S, C(S), and Y surfaces), each swept over 190 pairs of level‐set thresholds and then symmetrized under the 48‐fold cubic symmetry. The 103 HTAM structures were drawn from the Pareto‐optimal designs reported by Yeo et al. [16]. (Figure S2). The family counts reflect differences in accessible parameterization rather than an intentional balance, as discussed in the Results section.

Each unit cell was voxelated on a 64 × 64 × 64 grid using the Python library Trimesh, yielding a binary occupancy field of 262,144 voxels. Binary voxels encode solid and void regions in a form compatible with both finite‐element‐based property evaluation and machine‐learning‐based generative modeling. Cubic symmetry was then imposed to reduce redundant degrees of freedom, limit directional bias during screening, and enforce equivalent structural responses along the principal Cartesian directions. The full voxel grid was represented by an asymmetric unit under the 48 symmetry operations of the cubic point group (Figure 3A). Because voxels on symmetry boundaries are shared among multiple symmetry copies, the resulting number of independent voxels was 5984, corresponding to an effective numerical reduction of approximately 44‐fold rather than exactly 48‐fold. This symmetry constraint should be understood as a modeling choice that supports efficient discovery in a broad but structured design space.

The dataset was subsequently filtered to focus on the low‐density regime most relevant to lightweight design and to remove mechanically inefficient structures. Two screening criteria were applied. First, structures with relative density above 0.6 were excluded to remove near‐solid architectures outside the targeted lightweight regime. Second, the remaining candidates were required to satisfy *E*/*E_s_* > (ρ/ρ_*s*_)^2^, thereby excluding structures whose stiffness fell substantially below the scaling expected of mechanically efficient cellular solids. After filtering, 662 structures remained and constituted the initial training dataset, to which the FEM‐validated candidates were appended in each round. The training dataset comprised 590 TPMS‐based, 35 strut‐based, and 37 HTAM structures.

### 5.2 Mechanical‐Property Evaluation and Normalization

The effective elastic properties of each voxelated unit cell were evaluated using finite element analysis based on numerical homogenization. Owing to the imposed cubic symmetry, a single macroscopic strain state was sufficient to obtain the independent normal stiffness components needed for the [100] Young's modulus. A unit strain vector, ***ε*** ^app^ = [1, 0, 0, 0, 0, 0]^*T*^, was applied in Voigt notation, yielding the volume‐averaged stress components ${\bar{\sigma }}_{11}$, ${\bar{\sigma }}_{22}$, and ${\bar{\sigma }}_{33}$. These responses define *C*_11_ and *C*_12_, from which the effective Young's modulus in the [100] direction was calculated using the cubic elastic relation as follows:

$${\tilde{E}}_{\left[100\right]}=\frac{\left(C_{11}-C_{12}\right)\left(C_{11}+2C_{12}\right)}{C_{11}+C_{12}}$$

All effective properties, $\tilde{P}$, were normalized to relative properties, $\bar{P}$, by dividing by the corresponding property of the solid constituent, *P_s_*, such that $\bar{P}=\tilde{P}/P_{s}$. Relative density was defined as the solid volume fraction of the unit cell. This normalization isolates architectural efficiency from the absolute properties of the constituent material and enables direct comparison across designs. All effective elastic properties were evaluated in the linear‐elastic regime on voxel meshes of C3D8R elements with unit constituent modulus and Poisson ratio 0.33, under symmetric displacement boundary conditions.

### 5.3 Generative Latent Representation

A standard β‐VAE was trained to map voxelated unit cells to a continuous 32‐dimensional latent space. The encoder consisted of three blocks of 3D convolution with 32 filters, a 3 × 3 × 3 kernel, ReLU activation, batch normalization, and 2 × 2 × 2 max pooling, followed by a fully connected layer. The output layer produced the mean and log‐variance vectors of a Gaussian latent distribution, and the reparameterization trick was applied to sample a 32‐dimensional latent vector.

The decoder mirrored the encoder in reverse, using three blocks of 3D transposed convolution with 32 filters, a 3 × 3 × 3 kernel, ReLU activation, and 2 × 2 × 2 upsampling. A final transposed convolution layer with one filter and sigmoid activation produced a voxel‐occupancy probability field. The output was binarized using a threshold of 0.5 when binary voxel fields were required for finite‐element evaluation and Hamming‐distance calculation.

The loss function combined binary cross‐entropy reconstruction loss with a β‐weighted KL divergence term for latent regularization. A β‐annealing schedule was used to balance reconstruction fidelity and latent regularity. During initial training, β was increased linearly from 0.002 to 0.11 over 1,000 epochs. During active learning retraining, β was increased from 0.001 to 0.1 over the first 100 epochs, and the model was retrained for 500 epochs per round. All training used the Adam optimizer with a batch size of 64.

### 5.4 Property Predictors for Young's Modulus and Density

A feedforward neural network was trained as a supervised predictor for relative Young's modulus from the 32‐dimensional latent vector. The network consisted of three hidden layers with 128 units each and ReLU activation, followed by a single linear output unit. Both inputs and outputs were scaled using min–max normalization. The model was trained using the Adam optimizer with mean‐squared‐error loss for 100 epochs at a batch size of 32. For the validation analyses reported in the Results section, predictor performance was assessed on held‐out test data (10% of the structures, fixed random seed). After satisfactory predictive accuracy had been established, the modulus predictor used for final optimization was retrained on the full validated dataset so that all available structure–property information contributed to the search. Relative density was obtained using a decoder‐based density predictor rather than a separately trained neural‐network surrogate. Given a latent vector, the β‐VAE decoder reconstructed the voxel‐occupancy field, which was binarized using a threshold of 0.5. Relative density was then computed as the fraction of solid voxels in the full 64 × 64 × 64 unit cell.

### 5.5 Multi‐Objective Optimization

Multi‐objective optimization was performed using NSGA‐II with a (μ+λ) evolutionary strategy, implemented via the DEAP library. The initial population consisted of the latent vectors of all validated structures, encoded and scaled to the [0, 1] range using min‐max normalization. In each generation, λ = 100 offspring were produced. Crossover was performed using blend crossover (α = 0.5) with a probability of 0.7, and mutation used a Gaussian operator with mean 0, standard deviation 0.1, and per‐gene probability 0.2, applied with a probability of 0.2. The optimization ran for 100 generations per active learning round. A delta penalty was applied to enforce bound constraints, restricting all genes to the [0, 1] range.

Candidates whose predicted relative modulus exceeded the upper scaling bound *E > ρ* ^1.3^, set above the stiffness–density trend of the training set to screen out implausible surrogate extrapolations, were rejected within the fitness evaluation. Decoded structures were reduced to their largest connected component, with connectivity defined by shared faces, edges, or vertices (26‐neighborhood), before evaluation. Empty or degenerate candidates were assigned sentinel outputs and excluded from the dataset. Of the 900 candidates proposed across nine rounds, 797 returned usable FEM results and were appended to the training set.

The two objectives were maximization of the relative Young's modulus predicted by the supervised modulus predictor and minimization of relative density computed from the thresholded decoded voxel occupancy. After optimization, candidates outside the density window of the training set $\left(\bar{\rho }>0.6\right)$ were discarded before high‐fidelity evaluation.

### 5.6 Active Learning Loop

The active learning loop was performed for nine rounds. In the first round, the pre‐trained β‐VAE and a freshly trained modulus predictor were used without retraining. In each subsequent round, the following steps were performed. First, the β‐VAE was retrained on the expanded dataset, consisting of the initial structures and all previously validated candidates, for 500 epochs with beta annealing from 0.001 to 0.1. Second, a new supervised modulus predictor was trained on the updated latent vectors and their corresponding FEM‐evaluated Young's modulus values. Third, NSGA‐II was run for 100 generations to identify candidate Pareto‐optimal latent vectors. Fourth, small Gaussian noise with a scale of 0.1 was added to the optimized latent vectors before decoding to encourage structural diversity. Fifth, the decoded candidates were evaluated by finite‐element homogenization in ABAQUS to obtain reference effective properties. The validated results were then appended to the dataset for the next round. This iterative process progressively expanded the validated design domain while keeping the predictor and generative model calibrated to newly explored regions.

Optimization progress was tracked using complementary metrics in structure space and objective space. Minimum Hamming distance was computed retrospectively to quantify how far newly validated structures were from the existing validated dataset, whereas Pareto hypervolume quantified improvement of the stiffness–density frontier. The active learning process was continued until hypervolume gains became progressively smaller, indicating that frontier improvement was approaching saturation under the present design assumptions and model capacity.

### 5.7 Fabrication of Carbon Nano‐Architectures

The six selected designs (Gen32, Gen35, Gen37, Ini32, Ini35, Ini37) were patterned into 5 × 5 × 5 lattice geometries in Blender and then exported as an STL file for slicing. Slicing for two‐photon polymerization was performed using DeScribe and subsequently printed using a Nanoscribe Photonics Professional GT (Nanoscribe Gmbh, Leopoldshafen, Germany). Printing was performed with IP‐Dip2 photoresist with 17.5 mW (35%) laser power, 7500 µm/s scan speed, 0.2‐0.05 µm adaptive slicing in Z, and 0.1 µm hatching in Y. Printed geometries were developed using SU8 developer (propylene glycol monomethyl ether acetate, PGMEA) including 1 h in an initial development followed by 1 week in a fresh bath with a 5 mm stir bar at 150 rpm in the opposite side of the dish. This process enables extensive development of extremely small pores and internal volumes as confirmed by cross‐sectional focused ion beam milling (Figure S10). The samples were then washed with IPA for 2 h and gently air dried.

The samples were then subjected to carbothermal reduction pyrolysis using a vacuum furnace (0.7‐1.2 Torr) with the profile: 7.5°C/min to 300°C, hold for 1 h, 7.5°C/min to 900°C, hold for 5 h, cool to RT. The lattices were suspended using a pillar and spring support which decouples the structure from the substrate enabling isotropic shrinkage during reduction.

### 5.8 Nanomechanical Compression Testing

The nano‐architectures were subjected to quasi‐static uniaxial compression with SEM in situ imaging. A FemtoTools NMT04 nanoindenter with a 200mN load cell and a 20 µm diameter diamond flat punch (Oxford Instruments) was positioned in a field emission scanning electron microscope (FEI Versa 3D). In situ compression tests were performed at 14 nm s^−1^ ($\dot{\varepsilon }$ = 10^−3^ s^−1^) and accelerating voltage and current were intentionally selected to avoid any electron‐induced damage during video collection. Young's modulus was extracted as the slope during the linear portion of loading, after the initial 1% and up to the onset of nonlinearity, so that the fitting interval was chosen for each specimen rather than fixed in advance. Strength was the maximum stress preceding the first significant stress drop, defined as a drop exceeding 5% of the stress immediately before it. To account for the carbon sink‐in of the lattice into the support pillar, the Sneddon correction [32] was applied for all samples as a carbon sample indenting into a carbon support pillar according to:

$$\Delta x_{lattice}=\Delta x_{total}-\frac{\left(1-{\nu_{PyC}}^{2}\right)f}{2E_{PyC}\bar{\;\rho }\;d/\sqrt{\pi }}$$

where *f* is the maximum force during uniaxial compression, *d* is the side‐length of the lattice in XY, and *E_PyC_* = 22 GPa and ν_*PyC*_ = 0.28 are the modulus and Poisson ratio of the monolithic pyrolyzed carbon of the support pillar. The term for deformation of the indenter is null due to the exceptionally high modulus of diamond.

Average values and standard deviations of Young's modulus and strength were taken from four independently tested specimens for each condition, except Ini35 and Gen37 for which three specimens were tested. All specimens were printed and processed in a single batch to avoid the influence of any batch‐to‐batch variability in development or pyrolysis on the comparative values of Gen and Ini structures. Differences between each Gen and Ini pair were assessed with Welch's unequal‐variance *t*‐test on the individual specimens. The three density‐matched pairs and the two properties were specified in advance, so no multiplicity correction was applied. Lattice density was taken as the CAD relative density multiplied by the density of the pyrolytic carbon, 1.40 g cm^−^^3^ [29]. For the architecture efficiency in Figure 6L, the constituent modulus of the pyrolytic carbon in the lattice walls was inferred from an unweighted least‐squares fit through the origin of the six geometry‐mean Young's moduli to the corresponding FEM relative moduli, which gives 45.8 GPa with a 95% confidence interval of 41 to 51 GPa from the standard error of the slope with five degrees of freedom, following the procedure of Crook et al. [3]. This value exceeds the monolithic modulus used in the compliance correction above and lies within the range reported for pyrolytic carbon at sub‐micron feature sizes [3, 31]. A direct measurement of the constituent modulus at the wall thickness of these lattices was not available, and the modulus of pyrolytic carbon depends on feature size, so the constituent modulus was inferred from the lattices themselves, as has been done for plate nanolattices. Independently of that fit, the measured lattice moduli require a constituent modulus of at least 31 GPa through the Voigt bound alone, so the inferred value is bounded from below without reference to any model. Deformation and failure modes were recorded throughout the compression sequences to compare the mechanical behavior of the generated and initial architectures.

## Author Contributions

J.Y. performed conceptualization, data curation, formal analysis, investigation, methodology, software, validation, visualization, wrote the original draft, wrote, review and edited the final manuscript. P.S. performed formal analysis, investigation, visualization, wrote the original draft, wrote, review and edited the final manuscript. D.P. performed conceptualization, methodology, formal analysis, visualization, wrote, review and edited the final manuscript. J.G. performed formal analysis, methodology, wrote, review and edited the final manuscript. S.R. performed conceptualization, funding acquisition, project administration, supervision, wrote, review and edited the final manuscript.

## Funding

This work was supported by the National Research Foundation of Korea (NRF) grant (RS‐2025‐16070951 and RS‐2023‐00222166) and by the InnoCORE program (N10260002) funded by Ministry of Science and ICT, as well as BK21 FOUR Program by the Ministry of Education, Republic of Korea. P.S would like to thank the financial support of the Schmidt Science Fellowship, Schmidt Sciences, the Rhodes Trust, and the Natural Sciences and Engineering Research Council of Canada (NSERC).

## Conflicts of Interest

The authors declare no conflicts of interest.

## Supporting information




**Supporting File**: advs78140‐sup‐0001‐SuppMat.pdf.

## Data Availability

The data that support the findings of this study are available within the article and its Supporting Information. The voxelated unit‐cell dataset, the associated mechanical properties, and the code used for dataset generation, model training, and multi‐objective optimization are openly available in GitHub at https://github.com/jinwook‐yeo/yeo‐2026‐generative‐ai‐architected‐materials.
