# Supplementary material for: Enhancing Specific Stiffness of Nano‐Architected Materials Through Generative AI and Active Learning
Source: Adv Sci (Weinh). 2026 Oct 4:e78140. Online ahead of print. doi: 10.1002/advs.78140 (PMC13634470; doi:10.1002/advs.78140)
Supplement: Supplementary file 1 [file ADVS-9999-e78140-s001.pdf]

# Supporting Information

## Enhancing Specific Stiffness of Nano-Architected Materials through Generative AI and Active Learning

Jinwook Yeo *et al.*

*Corresponding author. Email: ryush@kaist.ac.kr, jrgreer@caltech.edu

**This PDF file includes:**

Figure S1 to S10
Table S1 and S2

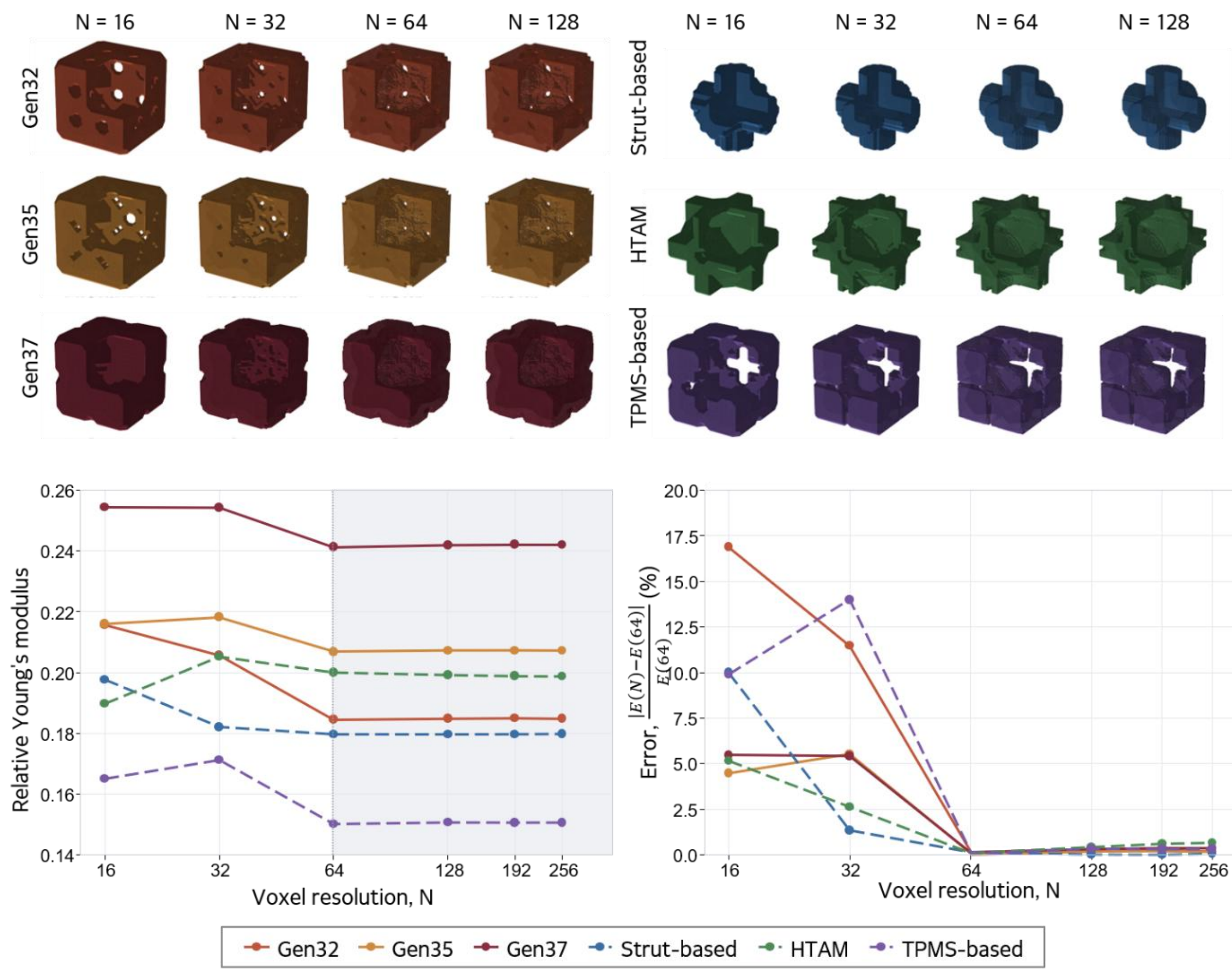


**Figure S1.**

Voxel-resolution sensitivity of the effective modulus. Top, the three optimized designs and one representative of each family (Ini35, strut-based; Ini37, HTAM; a TPMS-based member) as meshed at N = 16, 32, 64, and 128. Bottom left, relative Young's modulus against voxel resolution N. Bottom right, deviation from the value at N = 64. Shaded region, N ≥ 64.

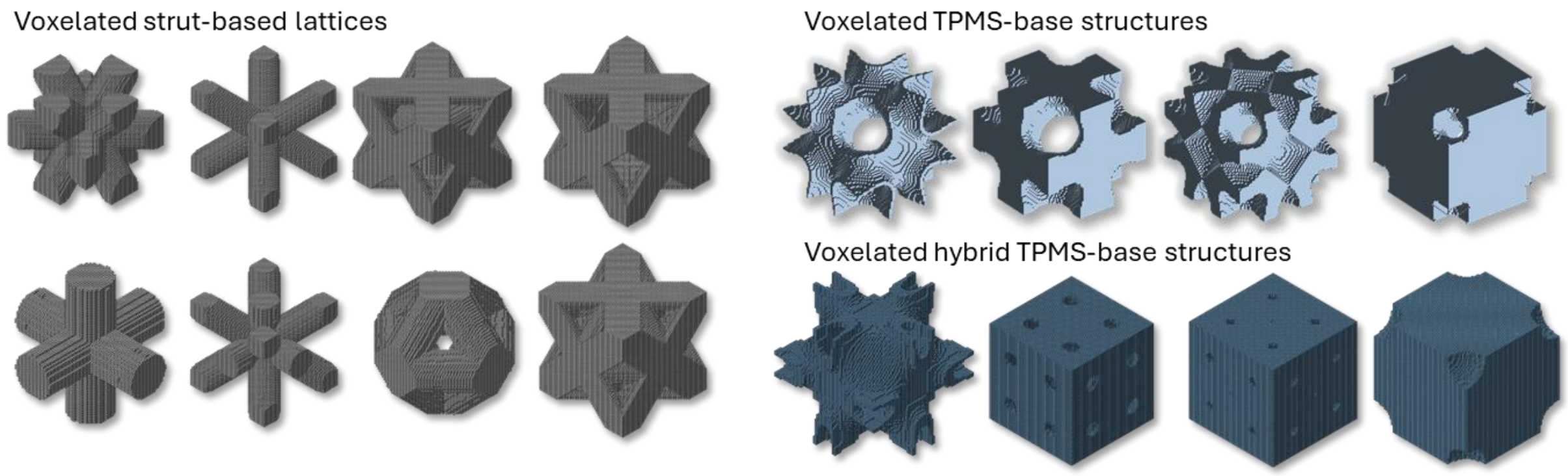


**Figure S2.**

The dataset comprises various architectural classes, including strut-based lattices (left), TPMS-based shells and volumes (right), and their hybrids, enabling the model to learn topological correlations across multiple material organization modes without geometric presupposition.

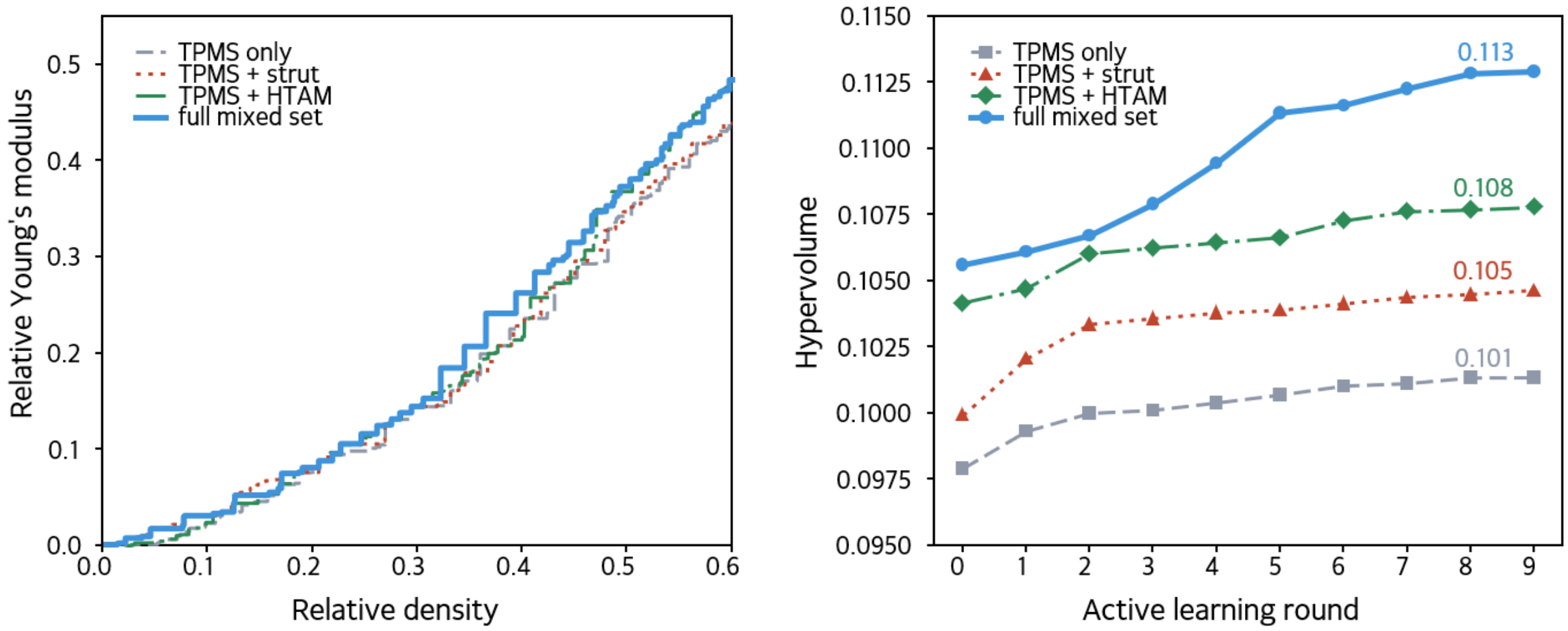


**Figure S3.**

Active learning started from subsets of the initial training dataset. (left) Cumulative Pareto fronts after nine rounds for searches started from the TPMS members alone, TPMS with strut members, TPMS with HTAM members, and the full mixed set (the reported run). (right) Pareto hypervolume of the cumulative front per round for the same four starts. Round 0, initial front of each start.

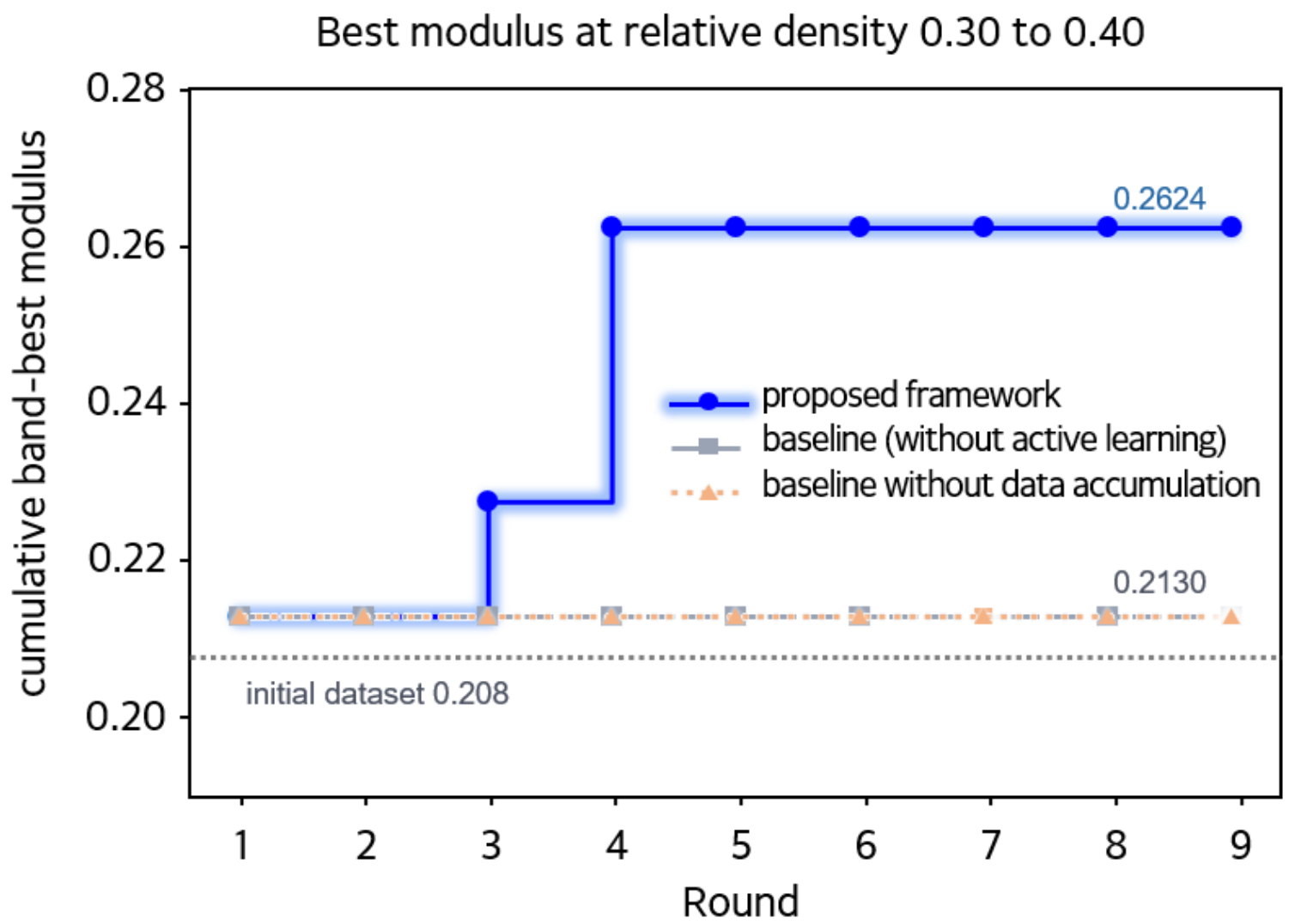


**Figure S4.**

Cumulative best relative Young's modulus within the relative density window 0.30 to 0.40 per active learning round for the proposed framework, the baseline without active learning, and the baseline without data accumulation. Dotted line, best member of the initial training dataset.

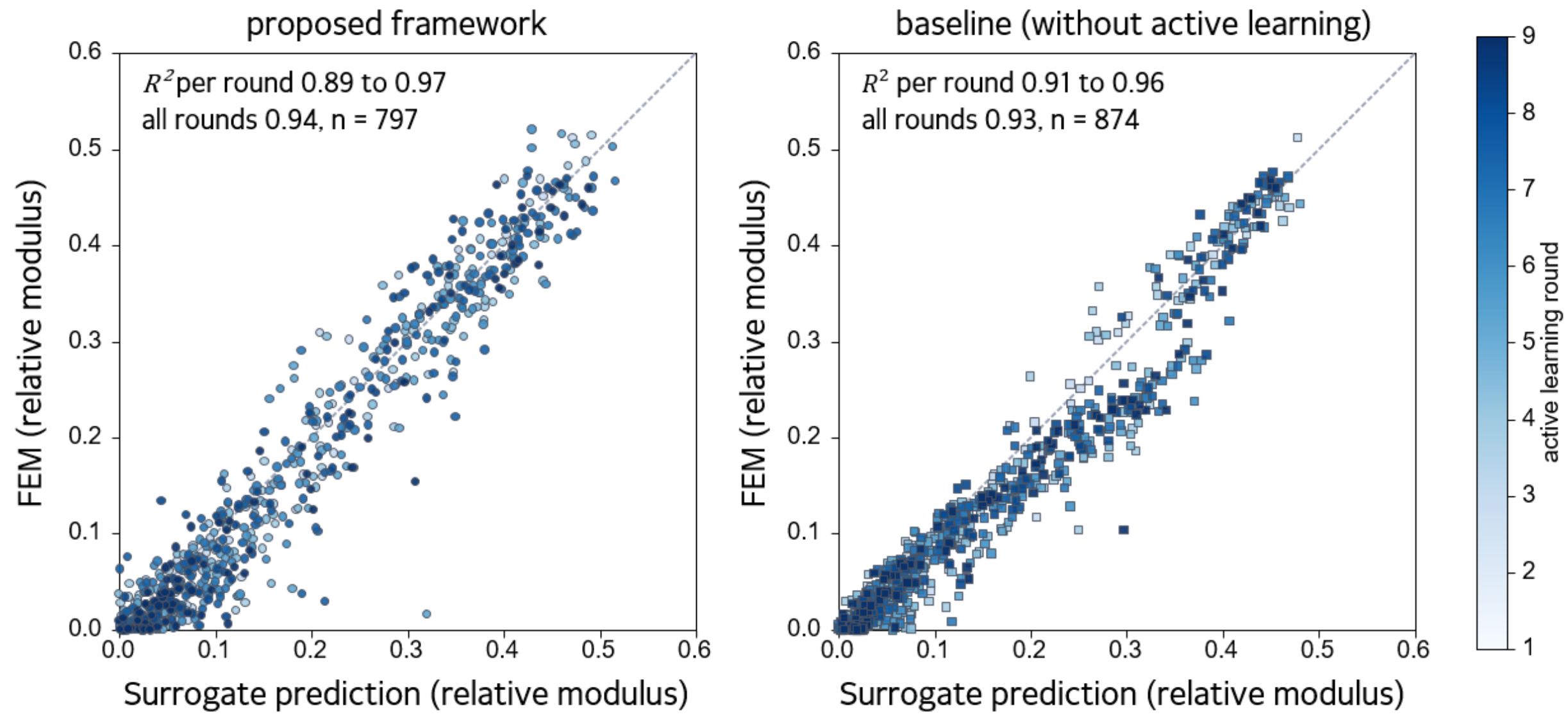


**Figure S5.**

Surrogate–FEM agreement for the candidates acquired by the proposed framework (left) and by the baseline without active learning (right). FEM relative modulus against the surrogate prediction, colored by active learning round; annotations give the per-round range and the pooled value of $R^2$.

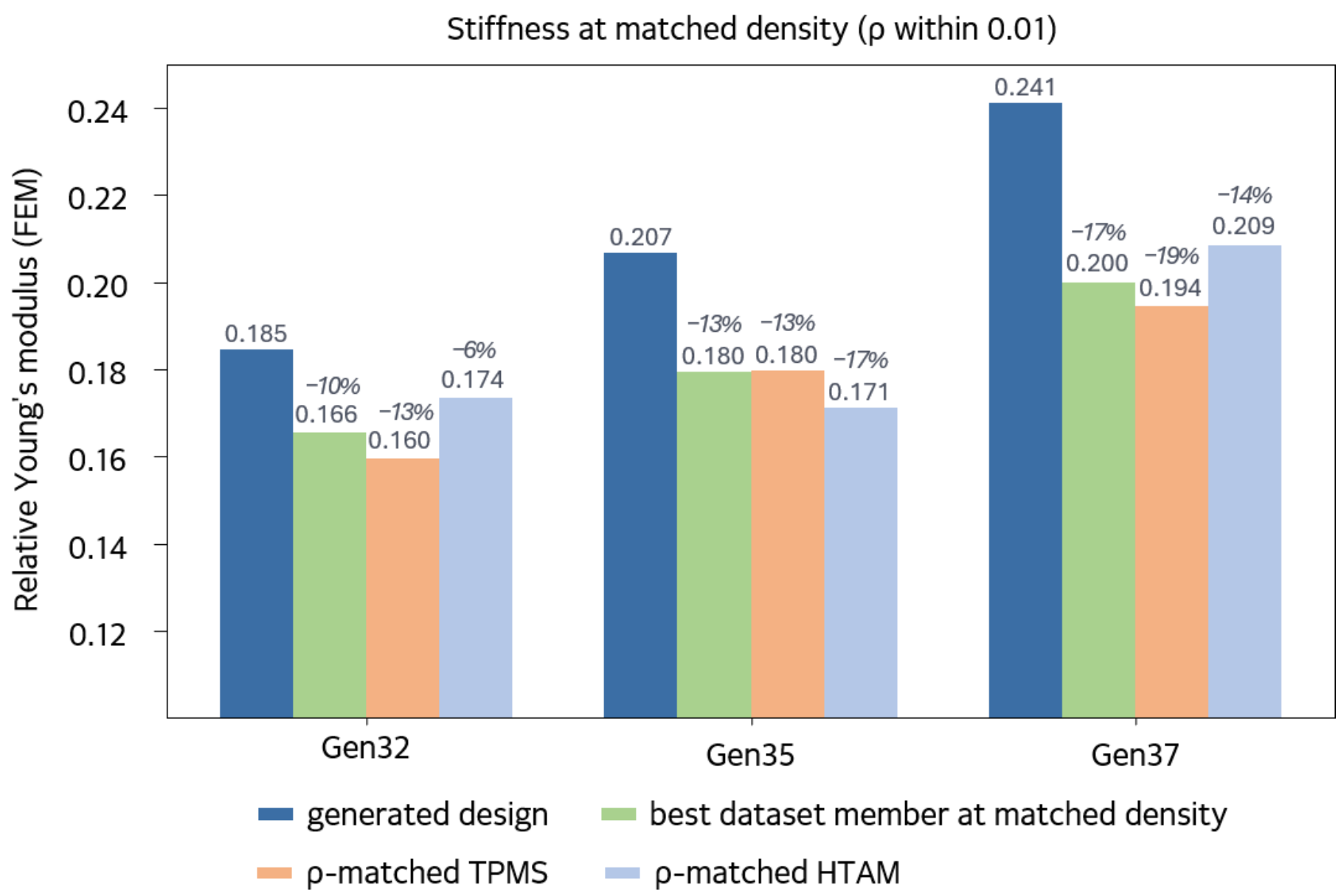


**Figure S6.**

Relative Young's modulus by FEM at matched relative density (within ±0.01) for each optimized design, the best initial-dataset member within the band, and the density-matched TPMS and HTAM fits. Percentages, difference from the optimized design.

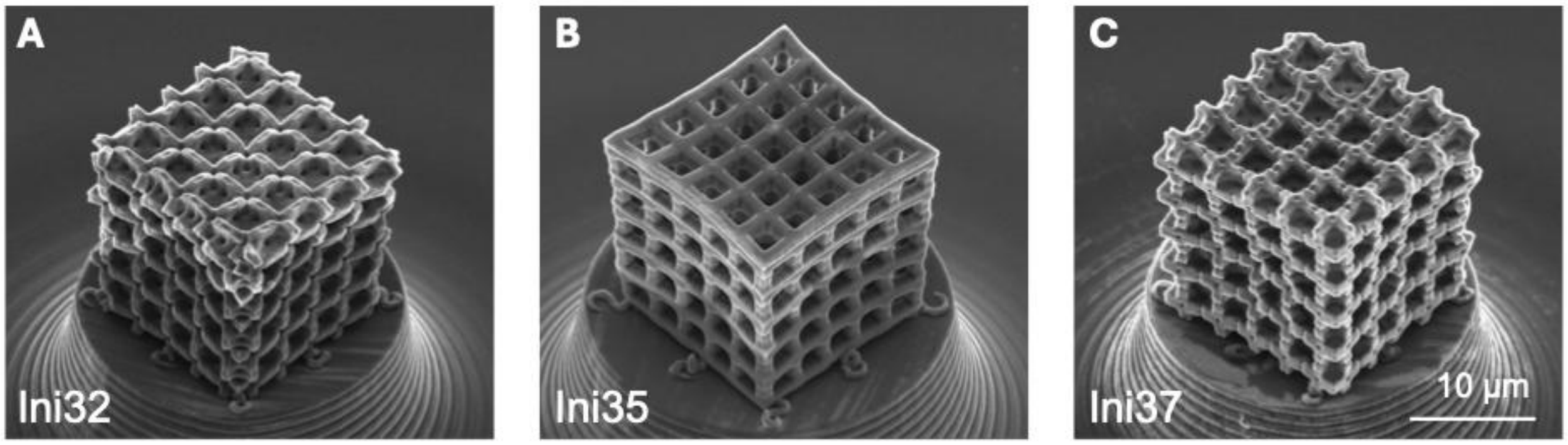


**Figure S7.**
Carbon nanolattices of the Ini structures (A) Ini32, (B) Ini35, and (C) Ini37

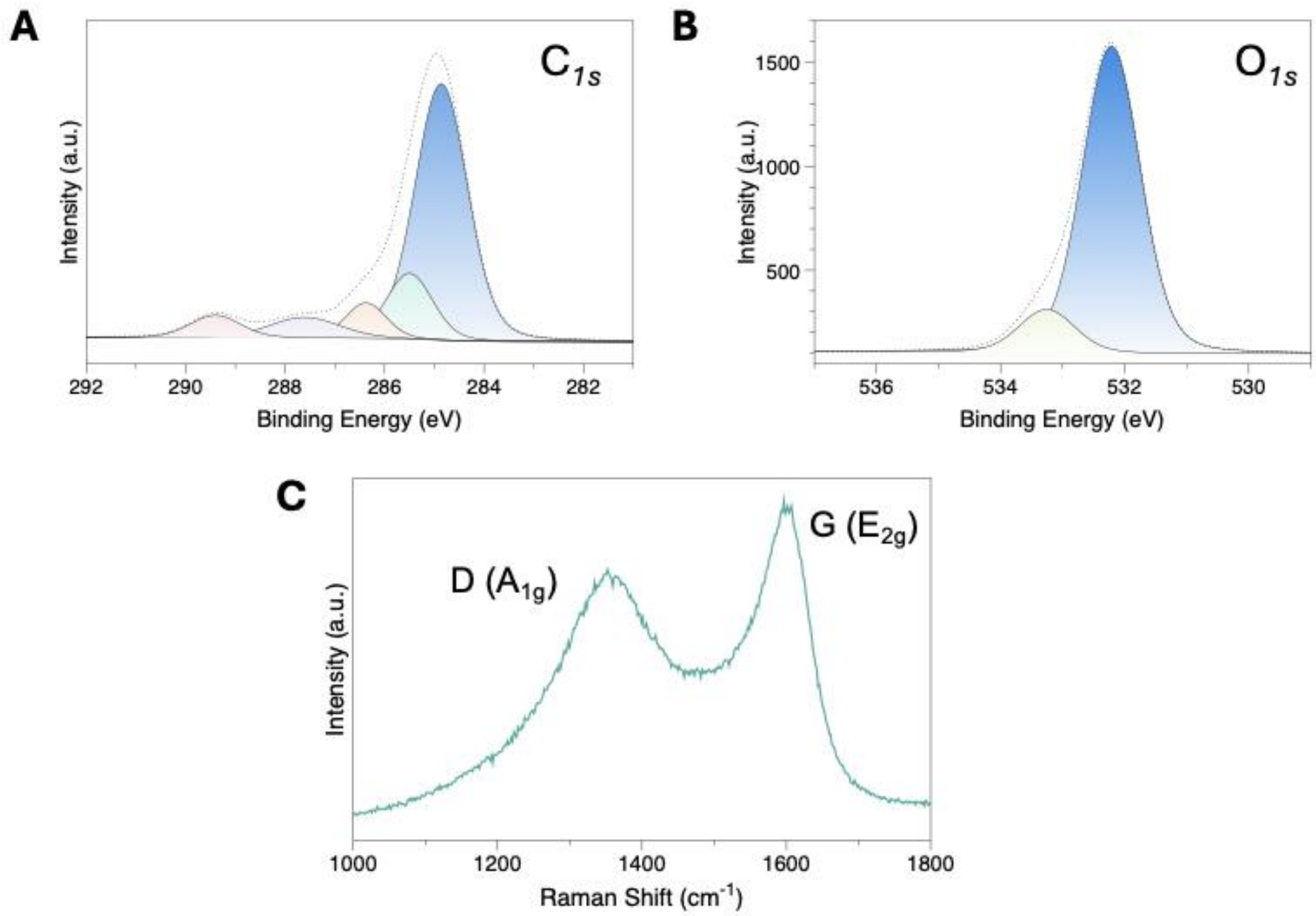


**Figure S8.**

Nanolattice carbon signatures (A) $C_{1s}$, (B) $O_{1s}$ X-Ray Photoelectron Spectroscopy (XPS) signatures, and (C) Raman Spectroscopy with the D and G bands.

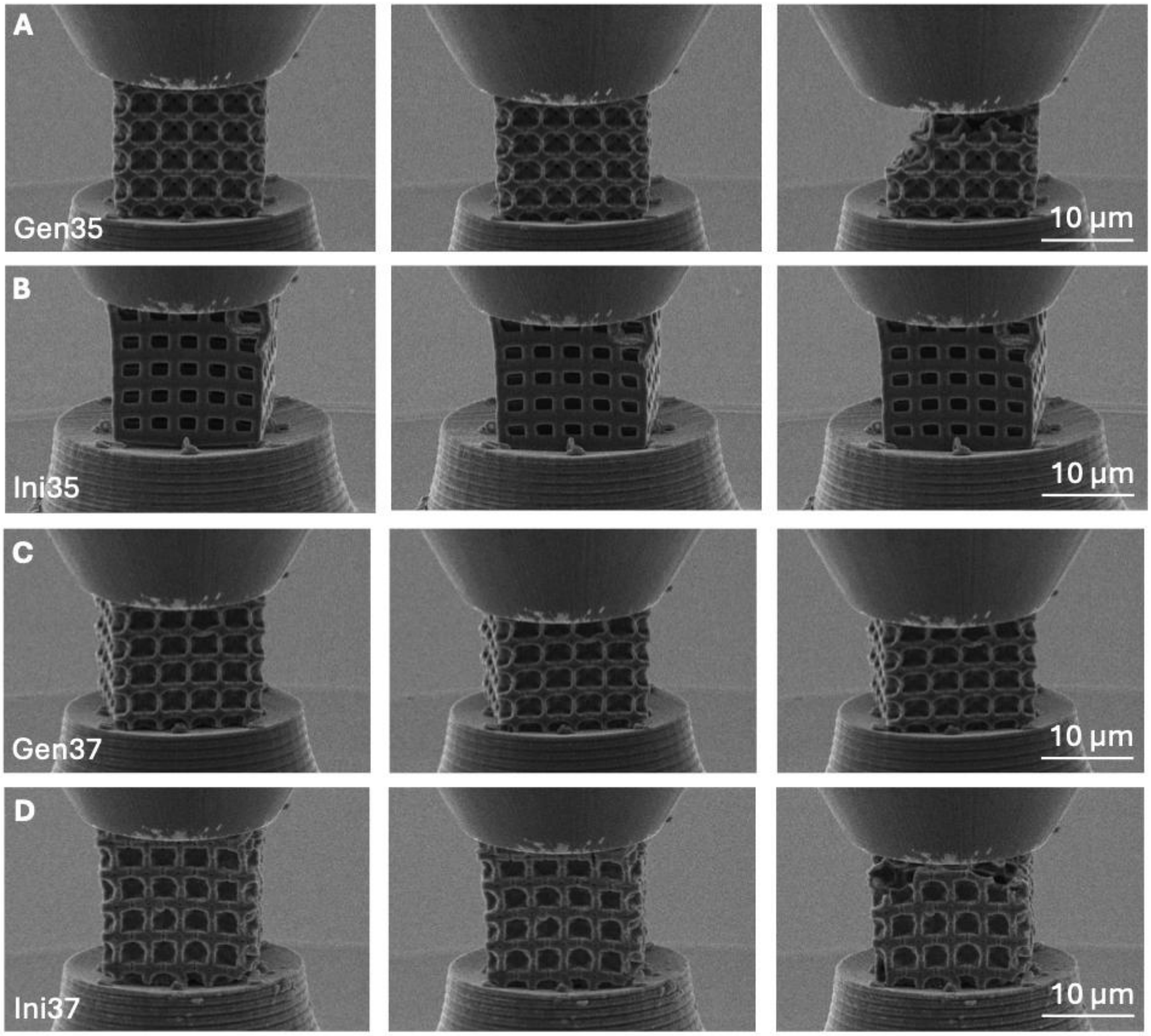


**Figure S9.**

In situ SEM image during compression of (A) Gen35, (B) Ini35, (C) Gen37, and (D) Ini37 nanoarchitectures at increasing strain (left to right).

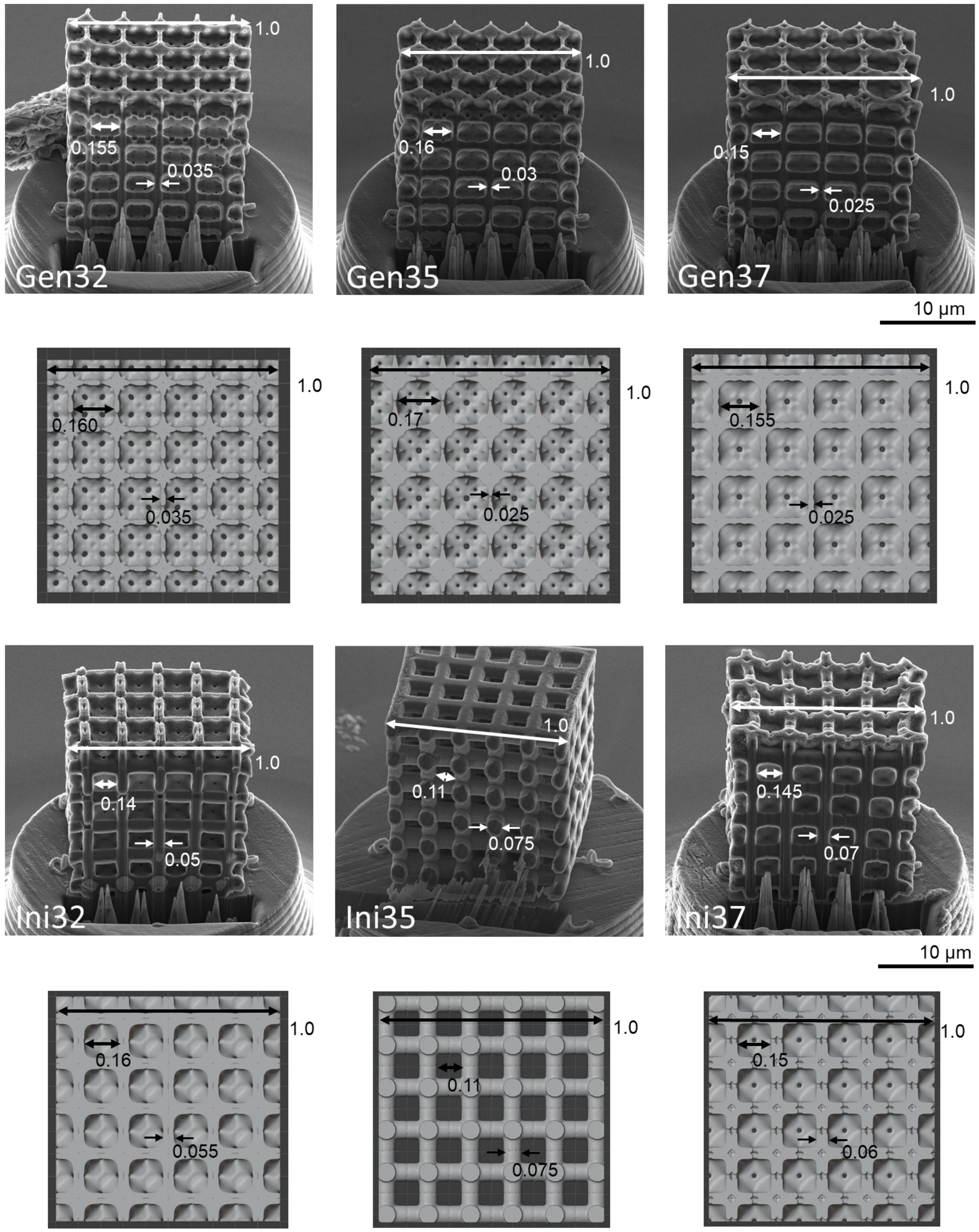


**Figure S10.**

Cross-sectional dimensions of the fabricated lattices compared with the CAD models. Top rows, FIB cross sections of Gen32, Gen35, and Gen37 and of Ini32, Ini35, and Ini37 with pore width and wall thickness normalized by the lattice width (1.0); bottom rows, the corresponding CAD cross sections with the same measurements. Scale bars, 10 µm. Dimensions are given in units of the outer width of the 5 × 5 × 5 lattice and are read to the nearest 0.005 of that width.

| | Relative Young's modulus | window | Initial Best Relative Young's modulus | gain |
|---|---|---|---|---|
| **Gen32** | 0.1845 | ±0.005 | 0.1656 (HTAM) | +11.4% |
| | | ±0.01 | 0.1656 (HTAM) | +11.4% |
| | | ±0.02 | 0.1669 (TPMS) | +10.5% |
| | | $\leq \rho_{Gen32}$ | 0.1529 (HTAM) | +20.7% |
| **Gen35** | 0.2068 | ±0.005 | 0.1796 (strut) | +15.2% |
| | | ±0.01 | 0.1796 (strut) | +15.2% |
| | | ±0.02 | 0.1942 (HTAM) | +6.5% |
| | | $\leq \rho_{Gen35}$ | 0.1669 (TPMS) | +23.9% |
| **Gen37** | 0.2412 | ±0.005 | 0.2000 (HTAM) | +20.6% |
| | | ±0.01 | 0.2004 (TPMS) | +20.4% |
| | | ±0.02 | 0.2079 (HTAM) | +16.1% |
| | | $\leq \rho_{Gen37}$ | 0.1942 (HTAM) | +24.2% |

**Table S1.**

Density-window benchmark of the optimized designs against the initial dataset (N = 3,675). For each design, the stiffest initial member within ±0.005, ±0.01, and ±0.02 relative density and among all members no denser than the design, with its family and the relative gain of the optimized design.

| Legend | Material and geometry summary | Reference |
|---|---|---|
| optimized designs (this work) | Pyrolytic carbon obtained by two-photon polymerization and pyrolysis. Generatively designed unit cells Gen32, Gen35, and Gen37 | this work |
| initial counterparts (this work) | Pyrolytic carbon obtained by two-photon polymerization and pyrolysis. Density-matched members of the initial dataset Ini32, Ini35, and Ini37 | this work |
| Al2O3 Octet Truss | Hollow Al2O3 shells created by two-photon polymerization and atomic layer deposition, followed by removal of the polymer template. Octet truss | S1 Meza et al., Science, 2014 |
| Au Foam | Nanoporous Au foam fabricated by electrochemical dealloying. Random nanoporous topology with about 25 nm ligaments | S2 Volkert et al., Appl. Phys. Lett., 2006 |
| Cu Foam | Nanoporous Cu foam fabricated by electrochemical dealloying. Random nanoporous topology with 20 to 115 nm cells | S3 Cheng and Hodge, Scripta Mater., 2013 |
| Cu Octet | Cu electroplated into a negative polymer template produced by direct laser writing. Octet lattice | S4 Gu and Greer, Extreme Mech. Lett., 2015 |
| Ni Double Gyroid | Ni electrodeposited into a self-assembled block-copolymer template. Two interpenetrating gyroids of opposite chirality | S5 Khaderi et al., Extreme Mech. Lett., 2017 |
| NiP Modified P-Surface | Electroless Ni-P thin film deposited on a polymer template. Shell-based modified Schwarz P surface | S6 Han et al., Adv. Mater., 2015 |
| NiP Octahedral | Electroless Ni-P deposition on a self-propagating photopolymer waveguide template. Hollow-tube octahedral unit cell | S7 Schaedler et al., Science, 2011 |
| Poly-Al2O3 Hex/Octet | Polymer scaffold written by two-photon polymerization and coated with Al2O3 by atomic layer deposition. Hexagonal and octet cells | S8 Bauer et al., Proc. Natl. Acad. Sci. U.S.A., 2014 |
| Poly-Al2O3 Honeycomb | Same polymer-ceramic composite as above. Honeycomb cells | S8 Bauer et al., Proc. Natl. Acad. Sci. U.S.A., 2014 |
| Poly-NiB Cubic Braced | Amorphous Ni-B coating deposited on a two-photon-polymerized polymer scaffold. Braced cubic unit cell | S9 Mieszala et al., Small, 2017 |
| Polymer Gyroid | Polymer TPMS lattice produced by additive manufacturing. Sheet-based gyroid | S10 Al-Ketan et al., Adv. Eng. Mater., 2018 |
| PyC Honeycomb | Glassy carbon obtained by pyrolysis of a two-photon-polymerized polymer. Honeycomb | S11 Bauer et al., Nat. Mater., 2016 |
| PyC Octet Truss (1) | Glassy carbon obtained by pyrolysis of a two-photon-polymerized polymer. Octet truss | S11 Bauer et al., Nat. Mater., 2016 |
| PyC-Al2O3 Octet Truss | Glassy carbon octet truss coated with Al2O3 | S11 Bauer et al., Nat. Mater., 2016 |
| PyC Octet Truss | Pyrolytic carbon octet truss | S11 Bauer et al., Nat. Mater., 2016 |
| PyC Isotropic Truss | Pyrolytic carbon obtained by pyrolysis of a two-photon-polymerized polymer. Isotropic truss | S12 Zhang et al., Proc. Natl. Acad. Sci. U.S.A., 2019 |
| PyC Octet Truss (2) | Pyrolytic carbon obtained by pyrolysis of a two-photon-polymerized polymer. Octet truss | S12 Zhang et al., Proc. Natl. Acad. Sci. U.S.A., 2019 |
| PyC Octet | Pyrolytic carbon octet | S12 Zhang et al., Proc. Natl. Acad. Sci. U.S.A., 2019 |

| Legend | Material and geometry summary | Reference |
|---|---|---|
| PyC I-WP Shell | Pyrolytic carbon shells obtained by pyrolysis of a two-photon-polymerized polymer. Sheet-based I-WP surface | S13 Wang et al., Proc. Natl. Acad. Sci. U.S.A., 2022 |
| PyC Neovius Shell | Pyrolytic carbon shells obtained by pyrolysis of a two-photon-polymerized polymer. Sheet-based Neovius surface | S13 Wang et al., Proc. Natl. Acad. Sci. U.S.A., 2022 |
| PyC Octahedral | Vitreous carbon obtained by pyrolysis of a self-propagating photopolymer waveguide lattice. Hollow octahedral cells | S14 Jacobsen et al., Carbon, 2011 |
| PyC Plate-Nanolattices | Pyrolytic carbon plate nanolattices obtained by two-photon polymerization and pyrolysis. Closed-cell plate architecture | S15 Crook et al., Nat. Commun., 2020 |
| PyC Shell Spinodal | Pyrolytic carbon spinodal shell nanoarchitecture obtained by two-photon polymerization and pyrolysis | S16 Guell Izard et al., Small, 2019 |
| Polymer-HEA Octet | High-entropy alloy film deposited on a two-photon-polymerized polymer octet lattice | S17 Zhang et al., Nano Lett., 2018 |
| Silica Inverse Opal | Inverse opal formed by colloidal self-assembly and atomic layer deposition of silica | S18 do Rosário et al., Extreme Mech. Lett., 2017 |
| Silica-TiO2 Inv. Opal | Inverse opal formed by colloidal self-assembly and atomic layer deposition of silica and TiO2 | S18 do Rosário et al., Extreme Mech. Lett., 2017 |
| SiOC Honeycomb | Polymer-derived SiOC ceramic from a preceramic resin. Honeycomb cells | S19 Eckel et al., Science, 2016 |
| SiOC Octahedral | Polymer-derived SiOC ceramic from a preceramic resin. Octahedral cells | S19 Eckel et al., Science, 2016 |
| SiOC Octet Truss | Polymer-derived SiOC nanolattices from a preceramic resin written by two-photon polymerization. Octet truss | S20 Bauer et al., Matter, 2019 |
| Ti-Alloy Octet Truss | Ti-6Al-4V octet truss assembled from laser-cut sheets and brazed | S21 Dong et al., Int. J. Solids Struct., 2015 |
| ZrNiAl Octahedron | Zr-Ni-Al metallic glass deposited on a two-photon-polymerized polymer template. Hollow octahedral cells | S22 Liontas and Greer, Acta Mater., 2017 |
| 316L BCC Shell | 316L stainless steel shell metamaterial of cubic symmetry produced by selective laser melting. Smooth-shell BCC | S23 Bonatti and Mohr, Acta Mater., 2019 |

**Table S2.**

Sources of the literature data in Figure 6J-L. For each legend entry, the constituent material, the fabrication route, and the unit-cell geometry, with the corresponding entry in the Supporting References. The material-class envelopes in (J) and (K) are taken from S24, and the bounds in (L) from S25.

## Supporting References


S1. L. R. Meza, S. Das, and J. R. Greer, "Strong, Lightweight, and Recoverable Three-Dimensional Ceramic Nanolattices," Science 345 (2014): 1322-1326.

S2. C. A. Volkert, E. T. Lilleodden, D. Kramer, and J. Weissmuller, "Approaching the Theoretical Strength in Nanoporous Au," Applied Physics Letters 89 (2006): 061920.

S3. I.-C. Cheng, and A. M. Hodge, "Strength Scale Behavior of Nanoporous Ag, Pd and Cu Foams," Scripta Materialia 69 (2013): 295-298.

S4. X. W. Gu, and J. R. Greer, "Ultra-Strong Architected Cu Meso-Lattices," Extreme Mechanics Letters 2 (2015): 7-14.

S5. S. N. Khaderi, M. R. J. Scherer, C. E. Hall, et al., "The Indentation Response of Nickel Nano Double Gyroid Lattices," Extreme Mechanics Letters 10 (2017): 15-23.

S6. S. C. Han, J. W. Lee, and K. Kang, "A New Type of Low Density Material: Shellular," Advanced Materials 27 (2015): 5506-5511.

S7. T. A. Schaedler, A. J. Jacobsen, A. Torrents, et al., "Ultralight Metallic Microlattices," Science 334 (2011): 962-965.

S8. J. Bauer, S. Hengsbach, I. Tesari, R. Schwaiger, and O. Kraft, "High-Strength Cellular Ceramic Composites With 3D Microarchitecture," Proceedings of the National Academy of Sciences 111 (2014): 2453-2458.

S9. M. Mieszala, M. Hasegawa, G. Guillonneau, et al., "Micromechanics of Amorphous Metal/Polymer Hybrid Structures With 3D Cellular Architectures: Size Effects, Buckling Behavior, and Energy Absorption Capability," Small 13 (2017): 1602514.

S10. O. Al-Ketan, R. Rezgui, R. Rowshan, H. Du, N. X. Fang, and R. K. Abu Al-Rub, "Microarchitected Stretching-Dominated Mechanical Metamaterials With Minimal Surface Topologies," Advanced Engineering Materials 20 (2018): 1800029.

S11. J. Bauer, A. Schroer, R. Schwaiger, and O. Kraft, "Approaching Theoretical Strength in Glassy Carbon Nanolattices," Nature Materials 15 (2016): 438-443.

S12. X. Zhang, A. Vyatskikh, H. Gao, J. R. Greer, and X. Li, "Lightweight, Flaw-Tolerant, and Ultrastrong Nanoarchitected Carbon," Proceedings of the National Academy of Sciences 116 (2019): 6665-6672.

S13. Y. Wang, X. Zhang, Z. Li, H. Gao, and X. Li, "Achieving the Theoretical Limit of Strength in Shell-Based Carbon Nanolattices," Proceedings of the National Academy of Sciences 119 (2022): e2119536119.

S14. A. J. Jacobsen, S. Mahoney, W. B. Carter, and S. Nutt, "Vitreous Carbon Micro-Lattice Structures," Carbon 49 (2011): 1025-1032.

S15. C. Crook, J. Bauer, A. Guell Izard, et al., "Plate-Nanolattices at the Theoretical Limit of Stiffness and Strength," Nature Communications 11 (2020): 1579.

S16. A. Guell Izard, J. Bauer, C. Crook, V. Turlo, and L. Valdevit, "Ultrahigh Energy Absorption Multifunctional Spinodal Nanoarchitectures," Small 15 (2019): 1903834.

S17. X. Zhang, Y. Yao, B. Wang, et al., "Three-Dimensional High-Entropy Alloy-Polymer Composite Nanolattices That Overcome the Strength-Recoverability Trade-Off," Nano Letters 18 (2018): 4247-4256.

S18. J. J. do Rosario, J. B. Berger, E. T. Lilleodden, R. M. McMeeking, and G. A. Schneider, "The Stiffness and Strength of Metamaterials Based on the Inverse Opal Architecture," Extreme Mechanics Letters 12 (2017): 86-96.

S19. Z. C. Eckel, C. Zhou, J. H. Martin, A. J. Jacobsen, W. B. Carter, and T. A. Schaedler, "Additive Manufacturing of Polymer-Derived Ceramics," Science 351 (2016): 58-62.
S20. J. Bauer, C. Crook, A. Guell Izard, et al., "Additive Manufacturing of Ductile, Ultrastrong Polymer-Derived Nanoceramics," Matter 1 (2019): 1547-1556.
S21. L. Dong, V. Deshpande, and H. Wadley, "Mechanical Response of Ti-6Al-4V Octet-Truss Lattice Structures," International Journal of Solids and Structures 60-61 (2015): 107-124.
S22. R. Liontas, and J. R. Greer, "3D Nano-Architected Metallic Glass: Size Effect Suppresses Catastrophic Failure," Acta Materialia 133 (2017): 393-407.
S23. C. Bonatti, and D. Mohr, "Smooth-Shell Metamaterials of Cubic Symmetry: Anisotropic Elasticity, Yield Strength and Specific Energy Absorption," Acta Materialia 164 (2019): 301-321.
S24. M. F. Ashby, Materials Selection in Mechanical Design, 4th ed., Butterworth-Heinemann: Oxford 2011.
S25. Z. Hashin, and S. Shtrikman, "A Variational Approach to the Theory of the Elastic Behaviour of Multiphase Materials," Journal of the Mechanics and Physics of Solids 11 (1963): 127-140.
